# Associations of *Batrachochytrium dendrobatidis* with skin bacteria and fungi on Asian amphibian hosts

**DOI:** 10.1038/s43705-023-00332-7

**Published:** 2023-11-22

**Authors:** Dan Sun, Jayampathi Herath, Shipeng Zhou, Gajaba Ellepola, Madhava Meegaskumbura

**Affiliations:** 1 Guangxi Key Laboratory for Forest Ecology and Conservation, College of Forestry, Guangxi University, Nanning, Guangxi 530000 People’s Republic of China; 2 School of Biomedical Sciences, International Institute of Health Sciences (IIHS), No 704 Negombo Rd, Welisara, 71722 Sri Lanka; 3 Department of Zoology, Faculty of Science, University of Peradeniya, Peradeniya, KY20400 Sri Lanka

**Keywords:** Microbial ecology, Evolution, Symbiosis, Fungal ecology

## Abstract

Amphibian skin harbors microorganisms that are associated with the fungal pathogen *Batrachochytrium dendrobatidis* (*Bd*), which causes chytridiomycosis, one of the most significant wildlife diseases known. This pathogen originated in Asia, where diverse *Bd* lineages exist; hence, native amphibian hosts have co-existed with *Bd* over long time periods. Determining the nuances of this co-existence is crucial for understanding the prevalence and spread of *Bd* from a microbial context. However, associations of *Bd* with the natural skin microbiome remain poorly understood for Asian hosts, especially in relation to skin-associated fungi. We used 16 S rRNA and fungal internal transcribed spacer (ITS) gene sequencing to characterize the skin microbiome of four native Asian amphibian species and examined the relationships between *Bd* infection and their skin bacterial and fungal communities; we also analyzed the correlates of the putative anti-*Bd* bacteria. We show that both skin bacterial and fungal community structure and composition had significant associations with infection status (*Bd* presence/absence) and infection intensity (frequency of *Bd* sequence reads). We also found that the putative anti-*Bd* bacterial richness was correlated with *Bd* infection status and infection intensity, and observed that the relative abundance of anti-*Bd* bacteria roughly correspond with changes in both *Bd* prevalence and mean infection intensity in populations. Additionally, the microbial co-occurrence network of infected frogs was significantly different from that of uninfected frogs that were characterized by more keystone nodes (connectors) and larger proportions in correlations between bacteria, suggesting stronger inter-module bacterial interactions. These results indicate that the mutual effects between *Bd* and skin-associated microbiome, including the interplay between bacteria and fungi, might vary with *Bd* infection in susceptible amphibian species. This knowledge will help in understanding the dynamics of *Bd* from a microbial perspective, potentially contributing to mitigate chytridiomycosis in other regions of the world.

## Introduction

The skin-associated microbiome is essential in protecting animals from disease-causing pathogens and this is apparent by their ability to outcompete pathogens for resources and produce antimicrobial compounds that are harmful to pathogens [1–4]. Amphibians, some of the first inhabitants of terrestrial ecosystems, possess a glandular skin that serves multiple functions. These include respiration, protection against predators, and defense against pathogens [5–7]. However, amphibians are also the most threatened group of vertebrates, with various factors contributing, including infectious diseases [8, 9]. Thus, understanding the relationship between amphibian skin diseases and their microbiome can offer insights to the disease ecology of amphibians.

The skin microbiota of amphibians is considered a primary defense mechanism against the chytrid fungus *Batrachochytrium dendrobatidis* (*Bd*), which causes chytridiomycosis, a widespread and serious amphibian disease [10–12]. *Bd* infection in amphibian skin alters the diversity and composition of the skin microbiota [13–16], thereby distrupting vital skin functions [17, 18]. Given this, the skin microbiome is thought to be crucial in preventing chytrid outbreaks [19, 20]. *In vitro* studies have indicated that numerous bacteria and several fungi present on hosts can inhibit *Bd* growth [5, 21, 22], while, *in vivo* studies have highlighted variability in composition and abundance of these anti-*Bd* microbiota across natural amphibian populations [23–25]. Furthermore, bacterial communities comprising multiple genera are known to offer more robust protection against *Bd* than a single strain [26, 27]. The interactions within microbial communities also influence host functionality, including defense against pathogens [28–31].

Interestingly, aside from a notable lethal outbreak in exotic frogs in Japan [11, 32, 33], there have not been reports of *Bd*-related amphibian mortality events or mass population declines in Asia. Whole-genome studies suggest East Asia as the probable region of origin for *Bd* and highlight it as an area for high *Bd* lineage diversity [34]. It is now thought that Asian amphibian hosts have coexisted with *Bd* for approximately 30 million years [35] and developed resistance to infections from both endemic and global *Bd* lineages [36, 37]. Studying the interactions between *Bd* and the skin microbiome can shed light as to why Asian amphibian hosts are resistant to this lethal pathogen. While previous research has provided insights into the skin bacterial diversity and compositions of various host species in relation to the pathogenic *Bd* in Asia [38–40], our understanding of the relationship between skin microbiota and this pathogen remains relatively limited for Asian hosts [39, 41]. Moreover, the significant under-sampling of fungi from Asian hosts hinders our understanding of the relationship between *Bd* and the fungal community.

In this study, we focused on four wild amphibian host species that inhabit the high-altitude forests of the Guangxi region in southern China. Previous studies have identified these species as *Bd*-positive, presenting with sub-clinical infections [42]. Guangxi, situated in southern China, is recognized as a region of high *Bd* lineage diversity, including both endemic and global pandemic lineages [34, 43, 44]. Thus, examining the skin-associated microbiota and *Bd* in southern China will help in understanding the ecological roles of the microbes and their hosts. We investigated the relationships between skin microbiota (both bacteria and fungi) and pathogenic *Bd* infection in Asian host species, considering factors like infection status (*Bd*-infected vs. *Bd*-uninfected) and infection intensity (measured by the number of *Bd* sequence reads). First, we assessed how *Bd* infection impacts the diversity and composition of the skin bacterial and fungal communities. Next, we analyzed the distribution patterns of potential anti-*Bd* bacteria on hosts and explored the correlation between *Bd* and the richness and relative abundance of these anti-*Bd* bacteria. Finally, for each host species, we contrasted the co-occurrence patterns of microbial communities in *Bd*-infected individuals with those in uninfected ones.

## Materials and methods

### Field sampling

We focused on four aquatic-breeding amphibian species from four widely divergent taxa, of which two were torrent dwelling stream breeders which have high possibility of *Bd* infection: Fujian metacarpal-tubercled toad (*Leptobrachella liui*) and Chungan torrent frog (*Amolops chunganensis*), and the other two being pond breeding arboreal species: Red-disked small treefrog (*Theloderma rhododiscus*) and Minimal treefrog (*Rhacophorus minimus*). The four species dwell in high-altitudinal forested areas in southern China and are documented as host species for *Bd* [42]. The adult individuals of the four species were collected (*N* = 63) between April and May 2021 during which pathogen prevalence is high, in Guangxi region, China (Table 1). Epidermal swabs were obtained from all individuals using sterile swabs and subsequently released at their captured locations [45]. We followed standardized protocols and biosecurity measures while collecting and analyzing the swabs to prevent cross contamination between individuals and transfer of pathogens across habitats. Ethical clearance was obtained from the Animal Care & Welfare Committee of Guangxi University (GXU2020-501).

**Table 1:** Sampling localities, total number of individuals and *Bd*-infected individuals per species.

Species	Individuals sampled	Locality	Infected individuals
*L. liui*	11	Shiliugongli	9
*A. chunganensis*	16	Hongtan	15
*T. rhododiscus*	21	Shiliugongli	8
*R. minimus*	15	Shiliugongli	11

### DNA extraction and microbial sequence processing

We extracted DNA from the swabs using DNeasy Blood and Tissue Kit (Qiagen, Hilden, Germany), which subsequently were amplified for genomic DNA using PCR.

The V4 region of the bacterial 16 S rRNA gene and the ITS2 region of the fungal internal transcribed spacer (ITS) were amplified (primers and PCR amplification conditions are in Table S1). PCR products were purified using Qiagen Gel Extraction Kit. Sequencing libraries were generated with NEBNext^®^ Ultra™ II DNA Library Prep Kit (NEB, Massachusetts, USA) according to the manufacturer’s recommendations. The library quality was evaluated using the Qubit^®^ 2.0 Fluorometer (Life Technologies, California, USA) and Agilent 2100 Bioanalyzer system (Agilent Technologies, California, USA). Finally, the library was sequenced on an Illumina NovaSeq platform (Novogene, Tianjin, China) and 250 bp paired-end reads were generated.

### Analysis of sequencing data

The 250 bp paired-end reads were filtered and processed with the QIIME2 pipeline [46]. Denoising, filtering, and chimera removal were performed with DADA2 [47], thus obtaining all initial amplicon sequence variants (ASVs) and their relative abundance in each sample, then filtered out the ASVs with abundance less than 5 in each sample. A total of 3.5 million and 3.8 million reads were retained for 16 S rRNA and ITS2 datasets, respectively.

Species annotation was performed using SILVA database v138.1 for bacteria [48] and UNITE v8.3 (10.05.2021) database for fungi [49]. We rarefied the least sequences per sample to reduce the effects of uneven sampling (25,192 for bacteria and 16,895 for fungi).

### Statistical analyses

#### Associations of alpha diversity to *Bd*

To evaluate skin bacterial and fungal diversity, we calculated the Shannon’s diversity index and ASV richness. We initially used nonparametric Wilcoxon tests to investigate whether alpha diversity values varied between *Bd*-infected and -uninfected individuals. However, as only a few individuals recorded absence of *Bd* in our samples - *A. chunganensis* (*N* = 1), *R. minimus* (*N* = 4), *L. liui* (*N* = 2), we could only use individuals of *T. rhododiscus* (*Bd*-: *N* = 13, *Bd*+: *N* = 8) for this comparison.

Second, we used species-specific linear models to observe the correlations between infection intensity (measured as numbers of *Bd* sequence reads) in infected individuals and alpha diversity values among species using “ggplot2” package [50] in R v4.2.0 [51]. Third, we constructed negative binomial generalized linear models to test for associations between infection intensity and alpha diversity indices for each species.

#### Associations of beta diversity to *Bd*

To investigate the dissimilarity of microbial community, we calculated the beta diversity based on weighted Bray-Curtis and unweighted Jacarrd distances, and stress values using the package “vegan” [52] and visualized distance correlations among samples using nonmetric multidimensional scaling (NMDS) plots. We examined whether *Bd* presence explained significant dissimilarity of skin bacterial and fungal communities of *T. rhododiscus*. We also examined whether beta diversity had significant relationships with *Bd* infection intensity throughout all species and for each species. All statistical significances were assessed by permutational multivariate analysis of variance (PERMANOVA) using the adonis2 function with 999 permutations.

#### Correlations of microbial taxa and *Bd*

To evaluate the significant associations of *Bd* with bacterial and fungal taxa, we investigated the relationships between *Bd* infection intensity and the relative abundance of classified microbial taxa and ASVs for each species using Pearson correlations with Benjamini-Hochberg as implemented using “psych” package [53]. We used the linear discriminant analysis (LDA) effect size (LEfSe) method as performed in the “microbiomeMarker” package [54–56], to compare the bacterial and fungal taxa that significantly accounted for differences between infected and uninfected individuals within *T. rhododiscus*.

#### Patterns of putative anti-*Bd* bacteria in wild hosts

To investigate the distribution of putative anti-*Bd* bacteria in the four host species, 100% matches were used to identify ASVs in our bacterial dataset to known anti-*Bd* isolates which constitute of antifungal properties based on a series of culturing, isolation and testing [57, 58] in Geneious v9.0.2 [59]. We compared and visualized the numbers of putative anti-*Bd* ASVs among the four amphibian species using the package “Vennerable” [60]. To examine the relationships between *Bd* presence/absence (*Bd*+ and *Bd*−), and the richness and relative abundance of anti-*Bd* bacteria, we performed binomial generalized linear models, but only included with *T. rhododiscus*. Subsequently, we ran negative binomial generalized linear models with *Bd*-infected individuals of all species and for each species included in the models. We also used Kruskal-Wallis test and Wilcoxon test with *p*-value correction to investigate whether the richness and relative abundance of anti-*Bd* bacteria varied across the four species, which showed different population-level infection prevalence and intensity.

### Co-occurrence networks

In order to compare the differences in associations among microbial communities between *Bd*-infected and *Bd*-uninfected frogs in *T. rhododiscus*, we used co-occurrence networks as it can reveal ecologically important correlations [61, 62]. For this, the bacterial and fungal ASVs presented in at least 80% out of all samples in each group with relative abundance > 0.01% were selected. To decrease the complexity in networks and to construct co-occurrence networks, spearman correlation coefficients (ρ) > 0.8 and adjusted *p*-values < 0.01, were used. We assessed the difference between networks based on bootstrapping node attributes (degree, between centrality, closeness centrality and transitivity) with 10,000 iterations. The node attributes between networks were compared by the two-sample Kolmogorov-Smirnov test using the ks.test function in the “stats” package. The network topological properties (the number of nodes and edges, average clustering coefficient, average degree, average path length, network diameter, graph density, and modularity), were calculated in the “igraph” package [63]. We visualized the co-occurrence networks using Gephi v0.9.7 [64] with a Fruchterman Reingold layout. To detect modules in co-occurrence networks, modularity (*M*) > 0.4 was selected [65]. Nodes were classified into four categories to identify their roles in networks based on their within-module connectivity (*Zi*) and among-module connectivity (*Pi*) [66]: Module hubs (*Zi* > 2.5), network hubs (*Zi* > 2.5 and *Pi* > 0.62), connectors (*Pi* > 0.62) and peripherals (*Zi* < 2.5 and *Pi* < 0.62) [67–69]. These network hubs, module hubs, and connectors can be defined as keystone nodes that play crucial roles in microbial community stability [70].

## Results

### Associations of alpha diversity to *Bd* infection

**Fig. 1:**
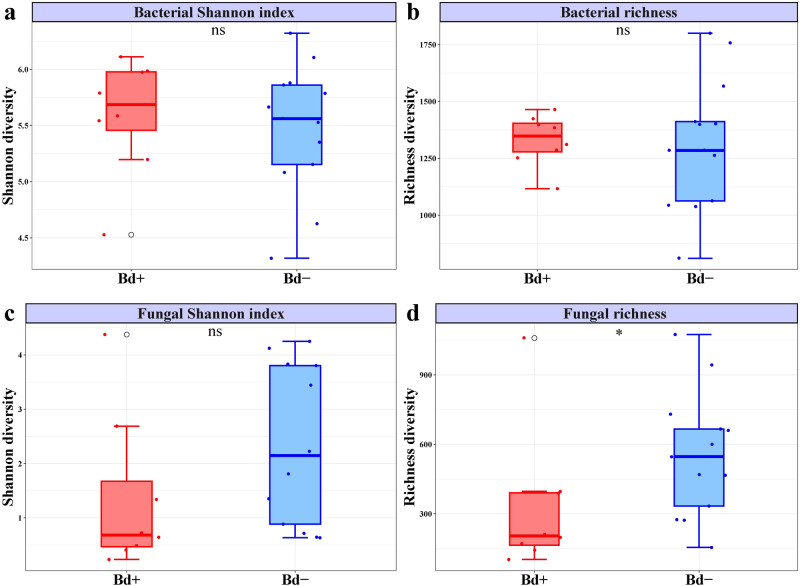
Skin bacterial and fungal diversity between infected and uninfected frogs. The comparison of bacterial diversity (**a**, **b**) and fungal diversity (**c**, **d**) between the *Bd*-infected and *Bd*-uninfected frogs of *T. rhododiscus*. The asterisk above plots indicates significant difference (Wilcox test, *p* < 0.05).

There was no significant difference in skin bacterial diversity between infected and uninfected frogs of *T. rhododiscus* (Fig. 1a, b). However, fungal Shannon index of infected frogs significantly differed from that of uninfected frogs, after removing an outlier (Fig. 1c). We also observed a significant reduction in fungal richness for infected frogs relative to uninfected frogs (Fig. 1d).

We found a significant correlation between bacterial richness and infection intensity for two species: *L. liui* (*p* = 0.028) and *T. rhododiscus* (*p* < 0.001). Shannon index for bacteria showed a positive relationship with infection intensity for *L. liui* (*p* = 0.011). However, both richness and Shannon index for fungi did not have significant correlations with infection intensity (Fig. S1).

### Associations of beta diversity to *Bd* infection

**Fig. 2:**
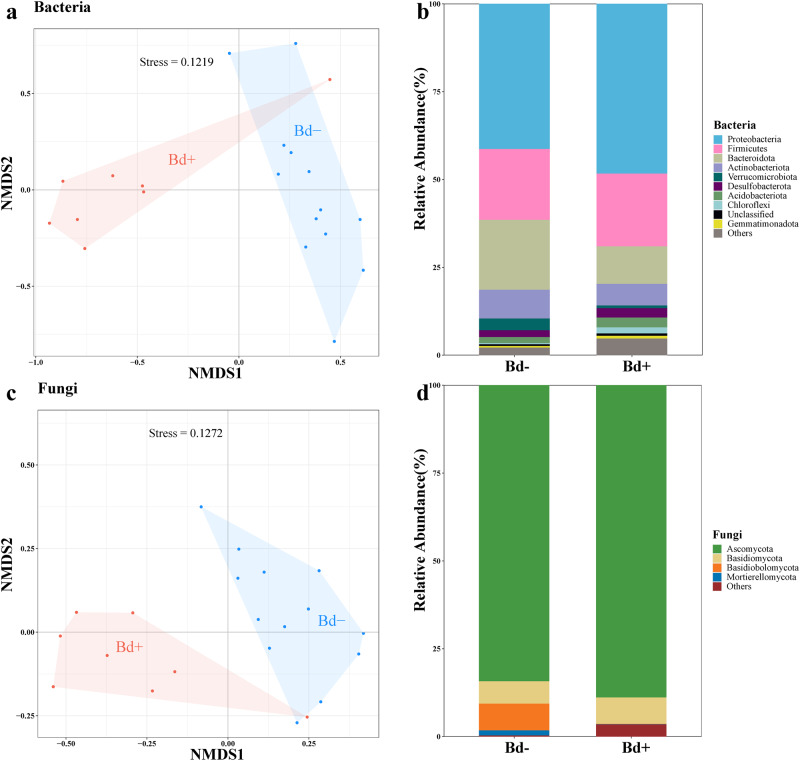
Skin microbiome compositions between infected and uninfected frogs. Skin bacterial community composition based on Bray-Curtis distance (**a**) and the relative abundance of the top ten dominated bacterial phyla (**b**); fungal community composition based on Jaccard distance (**c**) and the relative abundance of the top four fungal phyla among infected and uninfected individuals of *T. rhododiscus* (**d**). Each point in the NMDS stands for the values of Bray-Curtis or Jaccard distances for beta diversity of each frog’s skin bacterial or fungal communities.

We observed differences in the structure and composition of the skin bacterial and fungal communities between *Bd*-infected and uninfected frogs (Fig. 2). Notably, a significant difference was observed in the abundance-weighted composition of the bacterial community between infected and uninfected frogs (weighted Bray-Curtis PERMANOVA: *F* = 6.34, *R*^2^ = 0.25, *p* = 0.001; Fig. 2a). The phylum Proteobactria showed higher relative abundance whereas Bacteroidota showed lower relative abundance in infected frogs compared to uninfected frogs (Fig. 2b). The fungal community composition also showed a difference (unweighted Jaccard PERMANOVA: *F* = 1.55, *R*^2^ = 0.08, *p* = 0.001; Fig. 2c), with infected frogs exhibiting a lower abundance in the phylum Basidiobolomycota (Fig. 2d).

The bacterial and fungal community compositions significantly differed among the four species (Fig. S3); additionally, *Bd* infection intensity had a marginal influence on beta diversity of bacterial and fungal community (Table 2). Specifically, the presence-absence composition of the bacterial community differed significantly in infected individuals of *L. liui* (*p* = 0.021). With respect to fungal community composition, we detected significant correlations of infection intensity in *L. liui* and *T. rhododiscus* (Table S2).

**Table 2:** Results from weighted Bray-Curtis PERMANOVA analysis of skin microbial communities on the host species.

	Bacterial community			Fungal community		
	Pseudo-*F*	*p*-value	*R*^2^	Pseudo-*F*	*p*-value	*R*^2^
Infection intensity of *Bd*	2.800	0.001	0.034	2.402	0.004	0.031
Site	10.000	0.001	0.122	8.389	0.001	0.108
Species	5.373	0.001	0.131	4.097	0.001	0.106
*Bd* × Site	0.818	0.737	0.010	0.480	0.932	0.006
*Bd* × Species	1.221	0.105	0.030	1.429	0.009	0.037
Residuals			0.672			0.711

### Associations of bacterial and fungal taxa to *Bd* infection

**Fig. 3:**
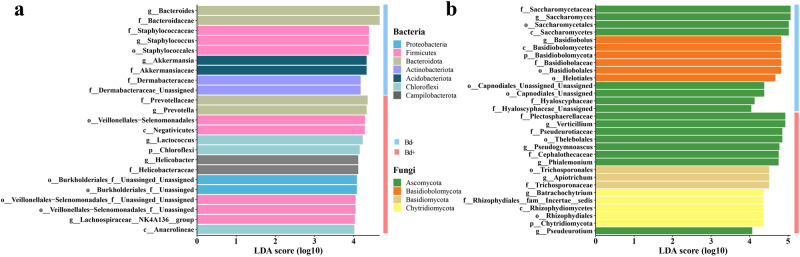
Biomarkers of skin bacteria and fungi between infected and uninfected frogs. Barplots show the bacterial (**a**) and fungal (**b**) taxonomic biomarkers between infected and uninfected individuals of the species *T. rhododiscus*.

We identified seven bacterial genera and eight fungal genera that are significantly related to *Bd* presence (Fig. 3). *Bd* infection intensity had significant correlations with the relative abundance of different bacterial and fungal taxa and ASVs on the four host species (Fig. S4; Table S3, S4). The bacterial genus *Prevotella* showed a positive correlation with infection intensity for both *A. chunganensis* and *T. rhododiscus* and were enriched in frogs infected with *Bd* (Fig. 3a). Furthermore, the bacterial genus *Bacteroides* and fungal order Helotiales were also positively correlated with infection intensity in *A. chunganensis* (Table S3, S4), but enriched in uninfected frogs of *T. rhododiscus* (Fig. 3b).

### Patterns of putative anti-*Bd* bacteria in wild hosts

**Fig. 4:**
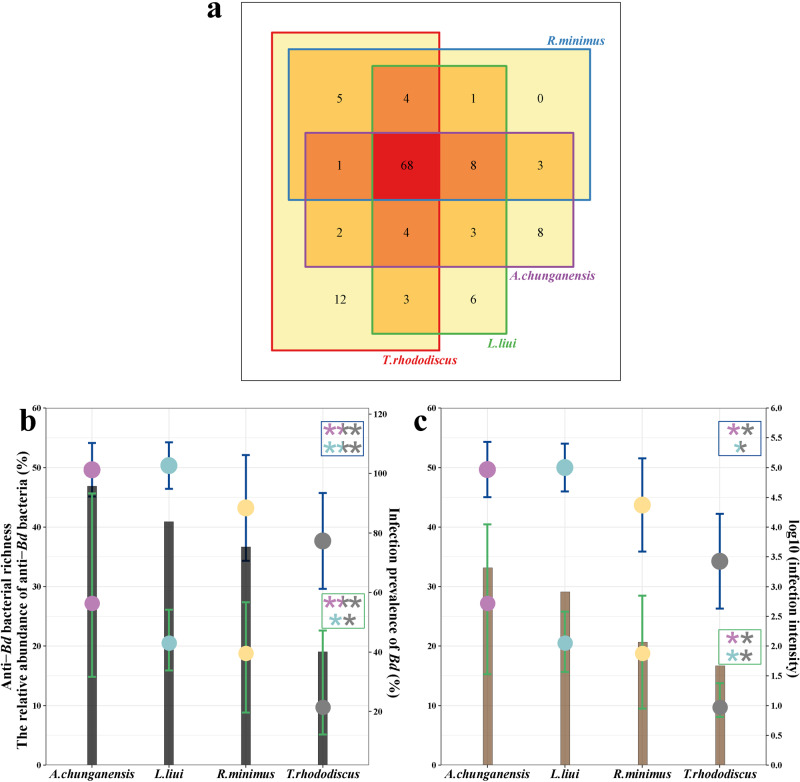
Putative anti-*Bd* bacteria on four amphibian species. Venn diagram shows the distributions of putative anti-*Bd* ASVs across the four host species reported positive to *Bd* infection (**a**). Putative anti-*Bd* bacterial richness and relative abundance across all individuals (**b**) and infected individuals (**c**). The left Y-axis and dots (colors represent each species) depict the richness (blue error bars) and relative abundance (green error bars) of putative anti-*Bd* bacteria. The right Y-axis and bars represent the prevalence of *Bd* and mean infection intensity for each population, respectively. The colored asterisks represent levels of significance between pairwise comparisons: ***p < 0.001, **p < 0. 01, *p < 0. 05.

From filtering the anti-*Bd* bacterial database, we found 127 putatively anti-*Bd* bacterial ASVs, exactly matching to those of anti-*Bd* isolates (Table S5). Of these, 68 ASVs were found to be common to all four host species (Fig. 4a), suggesting a potential shared mechanism for protection against *Bd* infection.

We detected a significant relationship between *Bd* presence/absence and the anti-*Bd* bacterial richness for *T. rhododiscus* (*p* = 0.02; Fig. S5). However, we did not observe a correlation between *Bd* presence/absence and the relative abundance of anti-*Bd* bacteria (*p* = 0.087). We also observed a significant positive relationship between infection intensity and anti-*Bd* bacterial richness in all infected individuals (belonging to four species) sampled (*p* = 0.005). *Bd* infection intensity was also positively correlated with the relative abundance of anti-*Bd* bacteria from *R. minimus* (*p* = 0.001) and *L. liui* (*p* = 0.004) as well.

At the population level, the richness and relative abundance of anti-*Bd* bacteria significantly differed among all individuals (Kruskal-Wallis test: *p* < 0.001 and *p* = 0.001) and among infected individuals of the four host species (Kruskal-Wallis test: *p* < 0.001 and *p* < 0.001). We observed that the relative abundance of putative anti-*Bd* bacteria correspond with changes in both the prevalence and mean infection intensity of *Bd* in host populations (Fig. 4b, c).

### Co-occurrence network of skin bacterial and fungal communities

**Fig. 5:**
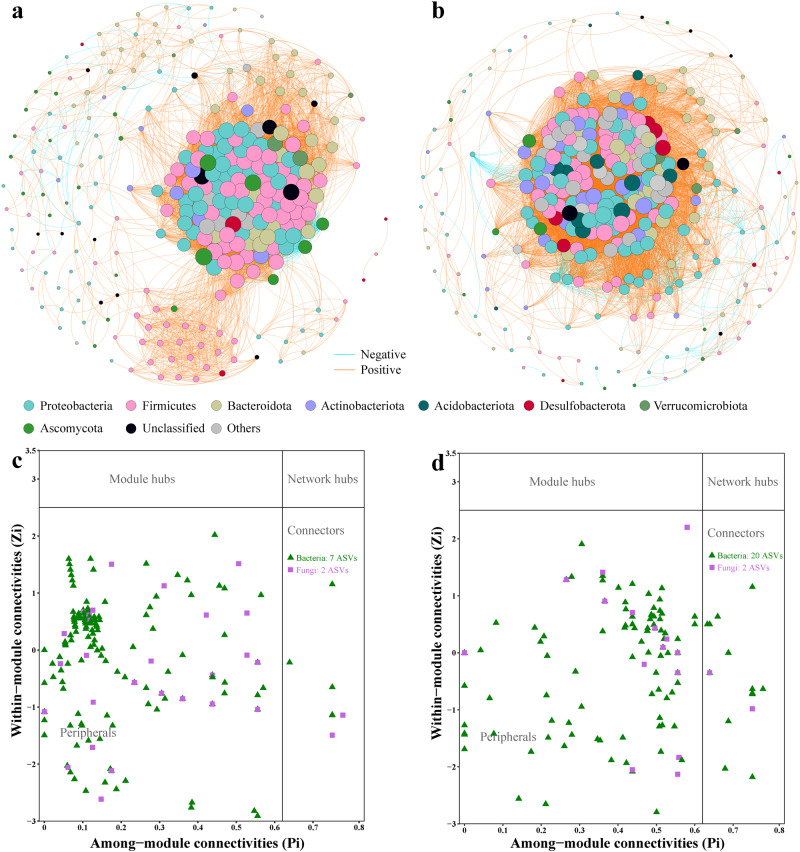
Microbial co-occurrence networks and putative keystone species. The co-occurrence networks of skin bacterial and fungal correlations on the *Bd*-uninfected frogs (**a**) and *Bd*-infected frogs (**b**). Putative keystone species of bacterial and fungal co-occurrence networks in *Bd*-uninfected frogs (**c**) and *Bd*-infected frogs (**d**). Nodes represent bacterial or fungal ASVs, and node size represents the degree of connectivity.

Our results revealed distinct differences in the correlations among the microbial communities between infected and uninfected frogs (Fig. 5; Table S6). The topological characteristics of the two co-occurrence networks are shown in Table S7. A higher clustering coefficient was found for uninfected frogs, representing a more complex network structure. The microbial co-occurrence network of infected frogs was characterized with lower average path length and diameter, indicating a compact network with strong interplays among microbial community.

For the microbial co-occurrence network of uninfected frogs, the most dominant members were from the bacterial phyla Firmicutes (34.74%), Proteobacteria (26.67%), Bacteroidota (16.14%), and fungal phylum Ascomycota (8.07%); the significant correlations of microbial taxa consisted of bacteria-bacteria (84.80%), bacteria-fungi (14.41%), and fungi-fungi (0.79%). A total of seven genera were classified as keystone nodes (connectors) in this network (Fig. 5c; Table S8).

In the co-occurrence network of microbial community on infected frogs, the most dominant members were from the bacterial phyla Proteobacteria (30.10%), Firmicutes (22.49%), Bacteroidota (14.88%) and Actinobacteriota (8.65%); the microbial relations consisted of bacteria-bacteria (95.24%), bacteria-fungi (4.63%), and fungi-fungi (0.13%). This network showed higher number of links and more connectors consisted of classified 19 genera (Fig. 5d; Table S8).

## Discussion

In recent years, the intricate relationship between amphibian skin microbiota and its protective role against pathogens has come under investigation. The skin-associated microbiota, in particular, has emerged as a potential shield against the deadly *Bd* infection [71], however information about the associations between skin microbiota and the pathogen *Bd* in Asian amphibian hosts with relatively low infection intensity is scarce. In this study, we assess the ecological traits of skin bacterial and fungal communities on four amphibian species to unravel the relationships between Asian host bacteria and fungi and *Bd* infection, and the potential protective role against this pathogen. We show that in Asian hosts, both bacterial and fungal community compositions are linked to *Bd* infection status and intensity. While bacterial diversity is influenced by infection intensity, fungal diversity is associated with infection status. Additionally, the distribution patterns of potential anti-*Bd* bacteria and the interplay between bacterial and fungal communities in infected versus uninfected frogs suggest that specific bacterial taxa and their interactions are crucial in defending against the lethal *Bd* infection. In the following sections we delve deeper into these findings, explaining the possible ways in which *Bd* shapes the amphibian skin microbiota across different Asian host species.

Our study showed the differential association of *Bd* presence/absence on fungal versus bacterial diversity. For instance, a marked decrease in fungal diversity was evident compared to bacterial diversity in the presence of *Bd*. Moreover, variations in *Bd* infection intensity showed an interesting correlation with bacterial diversity outcomes. In species like *L. liui* (stream breeding), a positive correlation was found between infection intensity and bacterial diversity, while the opposite was observed in *T. rhododiscus* (tree-hole breeding). These contrasting correlations might be attributed to the unique infection intensities experienced by different host species or perhaps due to the effects of host physiology and genetics [24, 72, 73]. This suggests that amphibian skin microbial communities may not have a common response to *Bd* infection; instead, they seem to vary considerably based on host-specific factors and the environments they inhabit. Therefore, there is a need for more extensive host-taxon sampling within amphibian populations and communities in Asia. Furthermore, given that skin microbiota could vary based on regional *Bd* lineages or specific strains [74], deeper analyses exploring the relationship between skin microbiota of native species and specific *Bd* populations is required.

Beyond the broad patterns in diversity, our results also showed specific associations between *Bd* and the microbial communities across the four studied host species. While most of these associations appeared correlative in nature, they strongly hint at the potential of *Bd* to influence the skin-associated microbiome of the host, leading to variations in both bacterial and fungal community structures and compositions. An intriguing outlier in our findings was the frog *T. rhododiscus*. Unlike the other seven infected frogs, its bacterial and fungal assemblages displayed distinct differences. This variation could possibly be attributed to an initial infection not exerting further influence on its skin microbial community, suggesting that the effects of *Bd* infections on the microbiome might be time-dependent [19, 72]. Comprehensive periodic future monitoring of bacterial and fungal populations in natural Asian hosts, coupled with controlled laboratory experiments measuring the impacts of infection intensity on microbial community dynamics, hence would provide invaluable insights. This would significantly deepen our understanding of the protective significance of skin microbiota throughout the course of *Bd* infection.

In the context of microbial community composition analyses, it is apparent that responses to *Bd* are associated with specific bacterial and fungal taxa present on the amphibian skin. In *T. rhododiscus*, certain enriched bacterial and fungal taxa appear to hold crucial roles in modulating *Bd* infection, potentially by affecting its colonization and proliferation given the resource competition within the skin microbiome [75]. Previous studies have shown the protective role of specific microbes; for instance, the genus *Bacteroides*, more abundant in uninfected frogs, is known to bolster host resistance to *Bd* by supporting gut homeostasis [76–78]. Similarly, uninfected frogs demonstrated greater fungal diversity and were enriched with anti-*Bd* bacteria like the genus *Staphylococcus* [79], reminiscent of patterns seen in tadpoles with enhanced immune functions [80]. Some microbes such as the bacterial genus *Prevotella* and *Sphingomonas*, however, showed positive correlations with *Bd* infection intensity, suggesting potential roles in influencing host sensitivity to *Bd*. It is imperative to note that our current sampling might not capture a comprehensive picture, indicating the necessity for more exhaustive research to elucidate the underlying mechanisms connecting *Bd* with these specific bacterial taxa.

Focusing on the associations between skin microbiota and *Bd*, we observed that anti-*Bd* bacteria is intricately linked with *Bd* infection status and intensity. This agrees with findings from previous research [20], which concluded that the richness and proportion of anti-*Bd* bacteria are directly correlated with *Bd* infection status and intensity in host species outside of Asia. On a population level, patterns in *Bd* prevalence and mean infection intensity mirrored variations in the relative abundance of these protective bacteria. Such patterns have been similarly observed in both American and Australian host species, further supporting the potential protective role of anti-*Bd* bacteria against clinical infections caused by *Bd* [81–83]. Yet, there remain anomalies; for instance, in certain American salamander species, populations with higher *Bd* infection prevalence paradoxically showed decreased levels of putative anti-*Bd* bacteria. This contrasting pattern might suggest that these particular hosts, like the salamanders, possess a unique tolerance to *Bd* [31].

We also noted co-occurrence patterns of skin microbial communities based on whether frogs are *Bd*-infected or -uninfected, suggesting a complex interplay between *Bd* infection and shifts in microbial interactions. In the microbial networks of uninfected frogs, there is a noticeable higher complexity, suggestive of a richer and more diverse microbial ecosystem. In contrast, the microbial network of infected frogs displayed stronger connector patterns, suggesting heightened inter-module communication [84]. These connectors, or keystone species, play a pivotal role in structuring the microbial community structure and function. An increased number of connectors may, in fact, bolster the stability and resilience of the microbial network in the face of *Bd* infections [67, 69, 85, 86]. However, our network analysis predominantly centered on a single susceptible species, emphasizing the urgency for broader sampling across more Asian host taxa.

## Conclusions

This study on amphibian skin microbiota and its protective functions against pathogens has highlighted the potential of skin-associated microbiota as a defense against *Bd* infection. Particularly in Asian amphibians, where multiple *Bd* lineages have co-existed with their hosts over long time scales, there is a noted gap in understanding this relationship. We examined the bacterial and fungal communities on four amphibian species, showing that both bacterial and fungal community compositions in Asian hosts relate to *Bd* infection status and intensity. Specifically, while bacterial diversity is affected by the infection intensity, fungal diversity correlates with the infection status. Distinct differences were observed among the species. For instance, *L. liui* displayed a positive correlation between infection intensity and bacterial diversity, whereas *T. rhododiscus* showed an opposite trend. These variations may be due to factors such as host physiology, genetics, and the habitats they occupy. Hence we emphasize that microbial responses to *Bd* infections may not be universal but rather influenced by host-specific factors. Furthermore, our work showed that *Bd* infection can influence the skin-associated microbiome of the host, causing variations in both bacterial and fungal community structures. Specific bacterial and fungal taxa on amphibian skin modulate *Bd* infection by affecting its colonization and proliferation, such as *Bacteroides* and *Staphylococcus*. However, some bacterial genera like *Prevotella* and *Sphingomonas* are positively correlated with *Bd* infection intensity. Our results also show that richness and proportion of anti-*Bd* bacteria correlate with *Bd* infection status and intensity. Furthermore, the microbial co-occurrence network in uninfected frogs is more complex, suggesting a diverse microbial ecosystem. In contrast, infected frogs show stronger connector patterns, indicating increased inter-module communication, which offer stability against *Bd* infections. However, we note that there is a need for broader sampling across more Asian host species to confirm these patterns.

### Supplementary information

Supplemental Materials Tables

Supplemental Materials Figures

## Data Availability

The raw 16S rRNA and ITS2 sequence data were deposited at the NCBI Sequence Read Archive under BioProject PRJNA943441.
