# Supplementary material for: Associations of Batrachochytrium dendrobatidis with skin bacteria and fungi on Asian amphibian hosts
Source: ISME Commun. 2023 Nov 22;3:123. doi: 10.1038/s43705-023-00332-7 (PMC10665332; doi:10.1038/s43705-023-00332-7)
Supplement: Supplementary file 1 — Supplemental Materials Tables [file 43705_2023_332_MOESM1_ESM.docx]

**Table S1.** Primers and PCR conditions used in this study.

| **Gene fragment** | **Primers** | **Region** | **Sequence (5’-3’)** | **PCR conditions** |
| --- | --- | --- | --- | --- |
| 16S rRNA | 515F | V4 | GTGCCAGCMGCCGCGGTAA | 1 min initial denaturation at 98 °C, 30 cycles at 98 °C of 10s, 50 °C of 30s, and 72 °C of 30s, with a final 5 min extension at 72 °C |
|  | 806R |  | GGACTACHVGGGTWTCTAAT |  |
| ITS | ITS3 | ITS2 | GCATCGATGAAGAACGCAGC | 1 min initial denaturation at 98 °C, 30 cycles at 98 °C of 10s, 50 °C of 30s, and 72 °C of 30s, with a final 5 min extension at 72 °C |
|  | ITS4 |  | TCCTCCGCTTATTGATATGC |  |

**Table S2.** Results from PERMANOVA analysis of skin fungal community on each species

|  |  | **Weighted Bray-Curtis** | | | **Unweighted Jaccard** | | |
| --- | --- | --- | --- | --- | --- | --- | --- |
|  | Species | Pseudo-*F* | *p*-value | R^2^ | Pseudo-*F* | *p*-value | R^2^ |
| All individuals | *A. chunganensis* | 1.227 | 0.199 | 0.081 | 0.987 | 0.489 | 0.658 |
|  | *L. liui* | 1.011 | 0.235 | 0.101 | 0.996 | 0.187 | 0.996 |
|  | *R. minimus* | 0.945 | 0.641 | 0.068 | 1.060 | 0.210 | 0.075 |
|  | *T. rhododiscus* | 0.477 | 0.408 | 0.024 | **1.113** | **0.026** | **0.055** |
| Infected individuals | *A. chunganensis* | 1.251 | 0.141 | 0.088 | 0.988 | 0.516 | 0.071 |
|  | *L. liui* | **1.457** | **0.019** | **0.172** | **1.097** | **0.028** | **0.135** |
|  | *R. minimus* | 0.981 | 0.504 | 0.098 | 1.070 | 0.189 | 0.106 |
|  | *T. rhododiscus* | 0.168 | 0.780 | 0.027 | 0.985 | 0.465 | 0.141 |

**Table S3.** Relative abundance of skin bacterial ASVs significantly correlated with *Bd* infection intensity among four host species.

| **Species** | **ASV** | **Phylum** | **Class** | **Order** | **Family** | **Genus** | **Perason's *r*** | ***p*-value** |
| --- | --- | --- | --- | --- | --- | --- | --- | --- |
| *T. rhododiscus* | ASV31 | Bacteroidota | Bacteroidia | Bacteroidales | Prevotellaceae | *Prevotella* | 0.970 | <0.001 |
| *T. rhododiscus* | ASV41 | Bacteroidota | Bacteroidia | Bacteroidales | Prevotellaceae | *Prevotella* | 0.974 | <0.001 |
| *T. rhododiscus* | ASV70 | Firmicutes | Bacilli | Lactobacillales | Streptococcaceae | *Lactococcus* | 0.747 | 0.033 |
| *T. rhododiscus* | ASV85 | Proteobacteria | Gammaproteobacteria | Enterobacterales | Yersiniaceae | Unassigned | 0.826 | 0.012 |
| *T. rhododiscus* | ASV86 | Firmicutes | Clostridia | Lachnospirales | Lachnospiraceae | *Agathobacter* | 0.810 | 0.015 |
| *T. rhododiscus* | ASV130 | Proteobacteria | Gammaproteobacteria | Pseudomonadales | Moraxellaceae | *Acinetobacter* | 0.790 | 0.020 |
| *T. rhododiscus* | ASV184 | Proteobacteria | Gammaproteobacteria | Burkholderiales | Comamonadaceae | Unassigned | -0.726 | 0.042 |
| *T. rhododiscus* | ASV205 | Firmicutes | Clostridia | Oscillospirales | Ruminococcaceae | *Faecalibacterium* | 0.884 | 0.004 |
| *T. rhododiscus* | ASV228 | Proteobacteria | Gammaproteobacteria | Burkholderiales | Sutterellaceae | *Parasutterella* | 0.821 | 0.012 |
| *T. rhododiscus* | ASV287 | Firmicutes | Clostridia | Oscillospirales | Ruminococcaceae | *Subdoligranulum* | 0.850 | 0.008 |
| *T. rhododiscus* | ASV299 | Bacteroidota | Bacteroidia | Bacteroidales | Prevotellaceae | *Prevotella* | 0.978 | <0.001 |
| *T. rhododiscus* | ASV447 | Actinobacteriota | Actinobacteria | Frankiales | Geodermatophilaceae | *Klenkia* | 0.787 | 0.020 |
| *T. rhododiscus* | ASV642 | Firmicutes | Negativicutes | Veillonellales-Selenomonadales | Selenomonadaceae | *Megamonas* | 0.790 | 0.020 |
| *T. rhododiscus* | ASV758 | Proteobacteria | Gammaproteobacteria | Burkholderiales | Comamonadaceae | Unassigned | -0.932 | 0.001 |
| *T. rhododiscus* | ASV817 | Firmicutes | Bacilli | Erysipelotrichales | Erysipelatoclostridiaceae | *Asteroleplasma* | 0.938 | 0.001 |
| *T. rhododiscus* | ASV1011 | Firmicutes | Clostridia | Lachnospirales | Lachnospiraceae | *Lachnospiraceae_NK4A136_group* | 0.923 | 0.001 |
| *T. rhododiscus* | ASV1343 | Bacteroidota | Bacteroidia | Bacteroidales | Prevotellaceae | *Alloprevotella* | 0.941 | <0.001 |
| *R. minimus* | ASV20 | Proteobacteria | Gammaproteobacteria | Enterobacterales | Hafniaceae | *Hafnia-Obesumbacterium* | 0.992 | <0.001 |
| *R. minimus* | ASV35 | Firmicutes | Bacilli | Erysipelotrichales | Erysipelatoclostridiaceae | *Erysipelatoclostridium* | 0.881 | <0.001 |
| *R. minimus* | ASV188 | Proteobacteria | Alphaproteobacteria | Rhizobiales | Beijerinckiaceae | *Methylobacterium-Methylorubrum* | 0.780 | 0.005 |
| *R. minimus* | ASV335 | Proteobacteria | Alphaproteobacteria | Sphingomonadales | Sphingomonadaceae | *Sphingomonas* | 0.623 | 0.041 |
| *R. minimus* | ASV921 | Firmicutes | Clostridia | Unassigned | Unassinged | Unassigned | 0.834 | 0.001 |
| *R. minimus* | ASV1193 | Firmicutes | Clostridia | Lachnospirales | Lachnospiraceae | Unassigned | 0.745 | 0.009 |
| *L. liui* | ASV23 | Proteobacteria | Gammaproteobacteria | Pseudomonadales | Pseudomonadaceae | *Pseudomonas* | 0.725 | 0.027 |
| *L. liui* | ASV176 | Gammaproteobacteria | Pseudomonadales | Pseudomonadaceae | Pseudomonas | Unassigned | 0.687 | 0.041 |
| *L. liui* | ASV275 | Proteobacteria | Gammaproteobacteria | Xanthomonadales | Xanthomonadaceae | Unassigned | 0.940 | <0.001 |
| *L. liui* | ASV291 | Proteobacteria | Alphaproteobacteria | Sphingomonadales | Sphingomonadaceae | Unassigned | 0.825 | 0.006 |
| *L. liui* | ASV392 | Firmicutes | Clostridia | Peptostreptococcales-Tissierellales | Peptostreptococcaceae | *Clostridioides* | 0.704 | 0.034 |
| *L. liui* | ASV473 | Bacteroidota | Bacteroidia | Bacteroidales | Prevotellaceae | *Prevotella* | 0.671 | 0.048 |
| *L. liui* | ASV514 | Proteobacteria | Alphaproteobacteria | Rhodobacterales | Rhodobacteraceae | *Rubellimicrobium* | 0.982 | <0.001 |
| *L. liui* | ASV550 | Firmicutes | Clostridia | Clostridiales | Clostridiaceae | Unassigned | -0.688 | 0.040 |
| *L. liui* | ASV649 | Gemmatimonadota | Longimicrobia | Longimicrobiales | Longimicrobiaceae | *Longimicrobium* | 0.668 | 0.049 |
| *L. liui* | ASV651 | Proteobacteria | Alphaproteobacteria | Rhizobiales | Rhizobiaceae | *Allorhizobium-Neorhizobium-Pararhizobium-Rhizobium* | 0.790 | 0.011 |
| *L. liui* | ASV853 | Actinobacteriota | Coriobacteriia | Coriobacteriales | Atopobiaceae | *Atopobium* | 0.820 | 0.007 |
| *L. liui* | ASV1101 | Actinobacteriota | Actinobacteria | Propionibacteriales | Nocardioidaceae | *Nocardioides* | -0.925 | <0.001 |
| *L. liui* | ASV1236 | Bacteroidota | Bacteroidia | Chitinophagales | Saprospiraceae | Unassigned | 0.753 | 0.019 |
| *A. chunganensis* | ASV7 | Bacteroidota | Bacteroidia | Bacteroidales | Bacteroidaceae | *Bacteroides* | 0.869 | <0.001 |
| *A. chunganensis* | ASV30 | Firmicutes | Clostridia | Peptostreptococcales-Tissierellales | Peptostreptococcaceae | *Romboutsia* | 0.731 | 0.002 |
| *A. chunganensis* | ASV31 | Bacteroidota | Bacteroidia | Bacteroidales | Prevotellaceae | *Prevotella* | 0.793 | <0.001 |

**Table S4.** Relative abundance of skin fungal ASVs significantly correlated with *Bd* infection intensity among four host species.

| **Species** | **ASV** | **Phylum** | **Class** | **Order** | **Family** | **Genus** | **Perason's *r*** | ***p*-value** |
| --- | --- | --- | --- | --- | --- | --- | --- | --- |
| *T. rhododiscus* | ASV28 | Ascomycota | Dothideomycetes | Pleosporales | Cucurbitariaceae | *Pyrenochaeta* | 0.987 | <0.001 |
| *T. rhododiscus* | ASV58 | Unassigned | Unassigned | Unassigned | Unassigned | Unassigned | 0.989 | <0.001 |
| *R. minimus* | ASV148 | Ascomycota | Sordariomycetes | Hypocreales | Unassigned | Unassigned | 0.871 | <0.001 |
| *L. liui* | ASV13 | Ascomycota | Leotiomycetes | Helotiales | Helotiaceae | *Sirococcus* | 0.999 | <0.001 |
| *L. liui* | ASV85 | Ascomycota | Dothideomycetes | Pleosporales | Didymellaceae | Unassigned | -0.673 | 0.047 |
| *L. liui* | ASV127 | Unassigned | Unassigned | Unassigned | Unassigned | Unassigned | 1.000 | <0.001 |
| *L. liui* | ASV145 | Ascomycota | Leotiomycetes | Helotiales | Helotiaceae | *Articulospora* | -0.703 | 0.035 |
| *L. liui* | ASV146 | Ascomycota | Dothideomycetes | Pleosporales | Unassigned | Unassigned | 1.000 | <0.001 |
| *L. liui* | ASV156 | Ascomycota | Dothideomycetes | Pleosporales | Didymellaceae | Unassigned | 0.990 | <0.001 |
| *L. liui* | ASV350 | Ascomycota | Unassigned | Unassigned | Unassigned | Unassigned | 0.879 | 0.002 |
| *L. liui* | ASV515 | Ascomycota | Eurotiomycetes | Eurotiales | Aspergillaceae | *Aspergillus* | 0.871 | 0.002 |
| *L. liui* | ASV537 | Ascomycota | Unassigned | Unassigned | Unassigned | Unassigned | 0.882 | 0.002 |
| *L. liui* | ASV601 | Ascomycota | Dothideomycetes | Capnodiales | Teratosphaeriaceae | *Devriesia* | 0.892 | 0.001 |
| *A. chunganensis* | ASV69 | Unassigned | Unassigned | Unassigned | Unassigned | Unassigned | 0.728 | 0.002 |
| *A. chunganensis* | ASV89 | Ascomycota | Sordariomycetes | Hypocreales | Nectriaceae | Unassigned | 0.704 | 0.003 |
| *A. chunganensis* | ASV106 | Ascomycota | Sordariomycetes | Xylariales | Xylariales_fam_Incertae_sedis | *Castanediella* | 0.623 | 0.013 |
| *A. chunganensis* | ASV119 | Ascomycota | Sordariomycetes | Hypocreales | Nectriaceae | *Volutella* | 0.843 | <0.001 |
| *A. chunganensis* | ASV273 | Basidiomycota | Microbotryomycetes | Microbotryomycetes_ord_Incertae_sedis | Chrysozymaceae | *Pseudohyphozyma* | 0.804 | <0.001 |
| *A. chunganensis* | ASV697 | Ascomycota | Unassigned | Unassigned | Unassigned | Unassigned | 0.762 | 0.001 |

**Table S5.** Putative anti-*Bd* ASVs among the four host species.

| **Species** | **ASV** | **Phylum** | **Class** | **Order** | **Family** | **Genus** | **Species** |
| --- | --- | --- | --- | --- | --- | --- | --- |
| *T. rhododiscus* | ASV1 | Proteobacteria | Gammaproteobacteria | Pseudomonadales | Pseudomonadaceae | *Pseudomonas* | Unassigned |
| *T. rhododiscus* | ASV3 | Proteobacteria | Gammaproteobacteria | Enterobacterales | Erwiniaceae | Unassigned | Unassigned |
| *T. rhododiscus* | ASV4 | Proteobacteria | Gammaproteobacteria | Enterobacterales | Enterobacteriaceae | Unassigned | Unassigned |
| *T. rhododiscus* | ASV6 | Proteobacteria | Gammaproteobacteria | Enterobacterales | Enterobacteriaceae | Unassigned | Unassigned |
| *T. rhododiscus* | ASV8 | Proteobacteria | Gammaproteobacteria | Pseudomonadales | Pseudomonadaceae | *Pseudomonas* | Unassigned |
| *T. rhododiscus* | ASV9 | Proteobacteria | Gammaproteobacteria | Burkholderiales | Oxalobacteraceae | *Janthinobacterium* | Unassigned |
| *T. rhododiscus* | ASV10 | Proteobacteria | Gammaproteobacteria | Pseudomonadales | Pseudomonadaceae | *Pseudomonas* | Unassigned |
| *T. rhododiscus* | ASV14 | Proteobacteria | Gammaproteobacteria | Pseudomonadales | Pseudomonadaceae | *Pseudomonas* | Unassigned |
| *T. rhododiscus* | ASV20 | Proteobacteria | Gammaproteobacteria | Enterobacterales | Hafniaceae | *Hafnia-Obesumbacterium* | Unassigned |
| *T. rhododiscus* | ASV23 | Proteobacteria | Gammaproteobacteria | Pseudomonadales | Pseudomonadaceae | *Pseudomonas* | Unassigned |
| *T. rhododiscus* | ASV28 | Proteobacteria | Gammaproteobacteria | Xanthomonadales | Xanthomonadaceae | *Stenotrophomonas* | rhizophila |
| *T. rhododiscus* | ASV37 | Proteobacteria | Gammaproteobacteria | Pseudomonadales | Pseudomonadaceae | *Pseudomonas* | Unassigned |
| *T. rhododiscus* | ASV48 | Proteobacteria | Gammaproteobacteria | Pseudomonadales | Pseudomonadaceae | *Pseudomonas* | Unassigned |
| *T. rhododiscus* | ASV52 | Proteobacteria | Gammaproteobacteria | Enterobacterales | Unassinged | Unassigned | Unassigned |
| *T. rhododiscus* | ASV57 | Bacteroidota | Bacteroidia | Flavobacteriales | Weeksellaceae | *Chryseobacterium* | piscium |
| *T. rhododiscus* | ASV67 | Actinobacteriota | Actinobacteria | Micrococcales | Microbacteriaceae | *Microbacterium* | Unassigned |
| *T. rhododiscus* | ASV76 | Proteobacteria | Gammaproteobacteria | Burkholderiales | Oxalobacteraceae | *Duganella* | Unassigned |
| *T. rhododiscus* | ASV77 | Proteobacteria | Gammaproteobacteria | Enterobacterales | Yersiniaceae | Unassigned | Unassigned |
| *T. rhododiscus* | ASV87 | Proteobacteria | Gammaproteobacteria | Enterobacterales | Unassinged | Unassigned | Unassigned |
| *T. rhododiscus* | ASV88 | Proteobacteria | Gammaproteobacteria | Aeromonadales | Aeromonadaceae | *Aeromonas* | Unassigned |
| *T. rhododiscus* | ASV98 | Proteobacteria | Gammaproteobacteria | Pseudomonadales | Moraxellaceae | *Acinetobacter* | Unassigned |
| *T. rhododiscus* | ASV103 | Proteobacteria | Gammaproteobacteria | Burkholderiales | Comamonadaceae | *Delftia* | Unassigned |
| *T. rhododiscus* | ASV105 | Proteobacteria | Gammaproteobacteria | Pseudomonadales | Pseudomonadaceae | *Pseudomonas* | Unassigned |
| *T. rhododiscus* | ASV108 | Proteobacteria | Gammaproteobacteria | Burkholderiales | Alcaligenaceae | *Alcaligenes* | Unassigned |
| *T. rhododiscus* | ASV114 | Proteobacteria | Gammaproteobacteria | Pseudomonadales | Pseudomonadaceae | *Pseudomonas* | Unassigned |
| *T. rhododiscus* | ASV116 | Proteobacteria | Gammaproteobacteria | Pseudomonadales | Pseudomonadaceae | *Pseudomonas* | Unassigned |
| *T. rhododiscus* | ASV130 | Proteobacteria | Gammaproteobacteria | Pseudomonadales | Moraxellaceae | *Acinetobacter* | Unassigned |
| *T. rhododiscus* | ASV145 | Bacteroidota | Bacteroidia | Flavobacteriales | Weeksellaceae | *Chryseobacterium* | Unassigned |
| *T. rhododiscus* | ASV149 | Proteobacteria | Gammaproteobacteria | Burkholderiales | Oxalobacteraceae | *Massilia* | Unassigned |
| *T. rhododiscus* | ASV152 | Actinobacteriota | Actinobacteria | Micrococcales | Micrococcaceae | Unassigned | Unassigned |
| *T. rhododiscus* | ASV166 | Proteobacteria | Gammaproteobacteria | Burkholderiales | Comamonadaceae | Unassigned | Unassigned |
| *T. rhododiscus* | ASV186 | Proteobacteria | Gammaproteobacteria | Burkholderiales | Comamonadaceae | Unassigned | Unassigned |
| *T. rhododiscus* | ASV197 | Bacteroidota | Bacteroidia | Sphingobacteriales | Sphingobacteriaceae | *Pedobacter* | Unassigned |
| *T. rhododiscus* | ASV202 | Proteobacteria | Gammaproteobacteria | Pseudomonadales | Pseudomonadaceae | *Pseudomonas* | Unassigned |
| *T. rhododiscus* | ASV209 | Proteobacteria | Gammaproteobacteria | Xanthomonadales | Rhodanobacteraceae | *Luteibacter* | Unassigned |
| *T. rhododiscus* | ASV215 | Proteobacteria | Alphaproteobacteria | Rhizobiales | Rhizobiaceae | *Allorhizobium-Neorhizobium-Pararhizobium-Rhizobium* | Unassigned |
| *T. rhododiscus* | ASV255 | Proteobacteria | Gammaproteobacteria | Pseudomonadales | Pseudomonadaceae | *Pseudomonas* | Unassigned |
| *T. rhododiscus* | ASV267 | Actinobacteriota | Actinobacteria | Micrococcales | Micrococcaceae | *Micrococcus* | Unassigned |
| *T. rhododiscus* | ASV274 | Proteobacteria | Gammaproteobacteria | Enterobacterales | Enterobacteriaceae | Unassigned | Unassigned |
| *T. rhododiscus* | ASV373 | Proteobacteria | Gammaproteobacteria | Pseudomonadales | Moraxellaceae | *Acinetobacter* | Unassigned |
| *T. rhododiscus* | ASV411 | Proteobacteria | Alphaproteobacteria | Caulobacterales | Caulobacteraceae | *Brevundimonas* | Unassigned |
| *T. rhododiscus* | ASV448 | Proteobacteria | Gammaproteobacteria | Pseudomonadales | Pseudomonadaceae | *Pseudomonas* | Unassigned |
| *T. rhododiscus* | ASV517 | Proteobacteria | Gammaproteobacteria | Enterobacterales | Unassinged | Unassigned | Unassigned |
| *T. rhododiscus* | ASV555 | Proteobacteria | Gammaproteobacteria | Enterobacterales | Enterobacteriaceae | Unassigned | Unassigned |
| *T. rhododiscus* | ASV561 | Proteobacteria | Gammaproteobacteria | Burkholderiales | Oxalobacteraceae | *Massilia* | Unassigned |
| *T. rhododiscus* | ASV571 | Bacteroidota | Bacteroidia | Flavobacteriales | Weeksellaceae | *Chryseobacterium* | Unassigned |
| *T. rhododiscus* | ASV590 | Proteobacteria | Gammaproteobacteria | Xanthomonadales | Xanthomonadaceae | *Stenotrophomonas* | Unassigned |
| *T. rhododiscus* | ASV591 | Actinobacteriota | Actinobacteria | Micrococcales | Microbacteriaceae | *Curtobacterium* | Unassigned |
| *T. rhododiscus* | ASV629 | Firmicutes | Bacilli | Exiguobacterales | Exiguobacteraceae | *Exiguobacterium* | Unassigned |
| *T. rhododiscus* | ASV660 | Proteobacteria | Gammaproteobacteria | Pseudomonadales | Moraxellaceae | *Acinetobacter* | Unassigned |
| *T. rhododiscus* | ASV707 | Proteobacteria | Gammaproteobacteria | Burkholderiales | Oxalobacteraceae | Unassigned | Unassigned |
| *T. rhododiscus* | ASV721 | Proteobacteria | Gammaproteobacteria | Enterobacterales | Enterobacteriaceae | Unassigned | Unassigned |
| *T. rhododiscus* | ASV732 | Proteobacteria | Gammaproteobacteria | Xanthomonadales | Xanthomonadaceae | *Stenotrophomonas* | Unassigned |
| *T. rhododiscus* | ASV768 | Proteobacteria | Gammaproteobacteria | Burkholderiales | Burkholderiaceae | *Burkholderia-Caballeronia-Paraburkholderia* | Unassigned |
| *T. rhododiscus* | ASV904 | Proteobacteria | Gammaproteobacteria | Burkholderiales | Oxalobacteraceae | *Duganella* | Unassigned |
| *T. rhododiscus* | ASV926 | Proteobacteria | Alphaproteobacteria | Rhizobiales | Rhizobiaceae | *Allorhizobium-Neorhizobium-Pararhizobium-Rhizobium* | Unassigned |
| *T. rhododiscus* | ASV957 | Proteobacteria | Gammaproteobacteria | Burkholderiales | Comamonadaceae | Unassigned | Unassigned |
| *T. rhododiscus* | ASV987 | Proteobacteria | Gammaproteobacteria | Burkholderiales | Comamonadaceae | Unassigned | Unassigned |
| *T. rhododiscus* | ASV988 | Firmicutes | Bacilli | Bacillales | Bacillaceae | *Bacillus* | Unassigned |
| *T. rhododiscus* | ASV1022 | Proteobacteria | Gammaproteobacteria | Xanthomonadales | Xanthomonadaceae | *Stenotrophomonas* | Unassigned |
| *T. rhododiscus* | ASV1023 | Bacteroidota | Bacteroidia | Flavobacteriales | Weeksellaceae | *Chryseobacterium* | Unassigned |
| *T. rhododiscus* | ASV1163 | Proteobacteria | Alphaproteobacteria | Rhizobiales | Rhizobiaceae | *Allorhizobium-Neorhizobium-Pararhizobium-Rhizobium* | Unassigned |
| *T. rhododiscus* | ASV1181 | Proteobacteria | Gammaproteobacteria | Pseudomonadales | Pseudomonadaceae | *Pseudomonas* | Unassigned |
| *T. rhododiscus* | ASV1308 | Bacteroidota | Bacteroidia | Flavobacteriales | Flavobacteriaceae | *Flavobacterium* | Unassigned |
| *T. rhododiscus* | ASV1313 | Firmicutes | Bacilli | Staphylococcales | Staphylococcaceae | *Staphylococcus* | Unassigned |
| *T. rhododiscus* | ASV1412 | Bacteroidota | Bacteroidia | Flavobacteriales | Flavobacteriaceae | *Flavobacterium* | Unassigned |
| *T. rhododiscus* | ASV1440 | Proteobacteria | Gammaproteobacteria | Burkholderiales | Comamonadaceae | *Comamonas* | Unassigned |
| *T. rhododiscus* | ASV1457 | Proteobacteria | Gammaproteobacteria | Pseudomonadales | Moraxellaceae | *Acinetobacter* | Unassigned |
| *T. rhododiscus* | ASV1526 | Proteobacteria | Gammaproteobacteria | Pseudomonadales | Pseudomonadaceae | *Pseudomonas* | Unassigned |
| *T. rhododiscus* | ASV1639 | Proteobacteria | Gammaproteobacteria | Burkholderiales | Oxalobacteraceae | *Massilia* | Unassigned |
| *T. rhododiscus* | ASV1642 | Proteobacteria | Alphaproteobacteria | Sphingomonadales | Sphingomonadaceae | *Novosphingobium* | Unassigned |
| *T. rhododiscus* | ASV1735 | Proteobacteria | Gammaproteobacteria | Pseudomonadales | Pseudomonadaceae | *Pseudomonas* | Unassigned |
| *T. rhododiscus* | ASV1807 | Bacteroidota | Bacteroidia | Sphingobacteriales | Sphingobacteriaceae | *Sphingobacterium* | Unassigned |
| *T. rhododiscus* | ASV1832 | Proteobacteria | Gammaproteobacteria | Xanthomonadales | Xanthomonadaceae | *Stenotrophomonas* | Unassigned |
| *T. rhododiscus* | ASV1935 | Actinobacteriota | Actinobacteria | Micrococcales | Microbacteriaceae | *Curtobacterium* | Unassigned |
| *T. rhododiscus* | ASV2395 | Bacteroidota | Bacteroidia | Flavobacteriales | Flavobacteriaceae | *Flavobacterium* | Unassigned |
| *T. rhododiscus* | ASV2459 | Actinobacteriota | Actinobacteria | Corynebacteriales | Nocardiaceae | *Rhodococcus* | Unassigned |
| *T. rhododiscus* | ASV2517 | Proteobacteria | Gammaproteobacteria | Pseudomonadales | Pseudomonadaceae | *Pseudomonas* | Unassigned |
| *T. rhododiscus* | ASV2816 | Proteobacteria | Gammaproteobacteria | Pseudomonadales | Pseudomonadaceae | *Pseudomonas* | Unassigned |
| *T. rhododiscus* | ASV3177 | Actinobacteriota | Actinobacteria | Streptomycetales | Streptomycetaceae | *Kitasatospora* | Unassigned |
| *T. rhododiscus* | ASV3254 | Actinobacteriota | Actinobacteria | Streptomycetales | Streptomycetaceae | *Streptomyces* | Unassigned |
| *T. rhododiscus* | ASV4216 | Proteobacteria | Gammaproteobacteria | Pseudomonadales | Pseudomonadaceae | *Pseudomonas* | Unassigned |
| *T. rhododiscus* | ASV4355 | Actinobacteriota | Actinobacteria | Micrococcales | Micrococcaceae | Unassigned | Unassigned |
| *T. rhododiscus* | ASV4379 | Proteobacteria | Gammaproteobacteria | Burkholderiales | Burkholderiaceae | *Burkholderia-Caballeronia-Paraburkholderia* | Unassigned |
| *T. rhododiscus* | ASV4637 | Actinobacteriota | Actinobacteria | Micrococcales | Micrococcaceae | *Paenarthrobacter* | Unassigned |
| *T. rhododiscus* | ASV5068 | Proteobacteria | Gammaproteobacteria | Xanthomonadales | Xanthomonadaceae | *Stenotrophomonas* | Unassigned |
| *T. rhododiscus* | ASV5169 | Proteobacteria | Gammaproteobacteria | Xanthomonadales | Xanthomonadaceae | *Lysobacter* | Unassigned |
| *T. rhododiscus* | ASV5454 | Firmicutes | Bacilli | Staphylococcales | Staphylococcaceae | *Staphylococcus* | Unassigned |
| *T. rhododiscus* | ASV6197 | Actinobacteriota | Actinobacteria | Micrococcales | Brevibacteriaceae | *Brevibacterium* | Unassigned |
| *T. rhododiscus* | ASV6951 | Proteobacteria | Gammaproteobacteria | Xanthomonadales | Xanthomonadaceae | Unassigned | Unassigned |
| *T. rhododiscus* | ASV7494 | Bacteroidota | Bacteroidia | Chitinophagales | Chitinophagaceae | *Terrimonas* | rubra |
| *T. rhododiscus* | ASV7569 | Proteobacteria | Gammaproteobacteria | Burkholderiales | Burkholderiaceae | *Burkholderia-Caballeronia-Paraburkholderia* | Unassigned |
| *T. rhododiscus* | ASV7678 | Proteobacteria | Alphaproteobacteria | Caulobacterales | Caulobacteraceae | *Brevundimonas* | terrae |
| *T. rhododiscus* | ASV8001 | Proteobacteria | Gammaproteobacteria | Pseudomonadales | Pseudomonadaceae | *Pseudomonas* | Unassigned |
| *T. rhododiscus* | ASV8883 | Firmicutes | Bacilli | Paenibacillales | Paenibacillaceae | *Paenibacillus* | Unassigned |
| *T. rhododiscus* | ASV9383 | Firmicutes | Bacilli | Paenibacillales | Paenibacillaceae | *Paenibacillus* | Unassigned |
| *T. rhododiscus* | ASV11664 | Actinobacteriota | Actinobacteria | Micrococcales | Microbacteriaceae | *Microbacterium* | Unassigned |
| *T. rhododiscus* | ASV19904 | Proteobacteria | Gammaproteobacteria | Aeromonadales | Aeromonadaceae | *Aeromonas* | Unassigned |
| *T. rhododiscus* | ASV22377 | Deinococcota | Deinococci | Deinococcales | Deinococcaceae | *Deinococcus* | Unassigned |
| *R. minimus* | ASV1 | Proteobacteria | Gammaproteobacteria | Pseudomonadales | Pseudomonadaceae | *Pseudomonas* | Unassigned |
| *R. minimus* | ASV3 | Proteobacteria | Gammaproteobacteria | Enterobacterales | Erwiniaceae | Unassigned | Unassigned |
| *R. minimus* | ASV4 | Proteobacteria | Gammaproteobacteria | Enterobacterales | Enterobacteriaceae | Unassigned | Unassigned |
| *R. minimus* | ASV6 | Proteobacteria | Gammaproteobacteria | Enterobacterales | Enterobacteriaceae | Unassigned | Unassigned |
| *R. minimus* | ASV8 | Proteobacteria | Gammaproteobacteria | Pseudomonadales | Pseudomonadaceae | *Pseudomonas* | Unassigned |
| *R. minimus* | ASV9 | Proteobacteria | Gammaproteobacteria | Burkholderiales | Oxalobacteraceae | *Janthinobacterium* | Unassigned |
| *R. minimus* | ASV10 | Proteobacteria | Gammaproteobacteria | Pseudomonadales | Pseudomonadaceae | *Pseudomonas* | Unassigned |
| *R. minimus* | ASV14 | Proteobacteria | Gammaproteobacteria | Pseudomonadales | Pseudomonadaceae | *Pseudomonas* | Unassigned |
| *R. minimus* | ASV20 | Proteobacteria | Gammaproteobacteria | Enterobacterales | Hafniaceae | *Hafnia-Obesumbacterium* | Unassigned |
| *R. minimus* | ASV23 | Proteobacteria | Gammaproteobacteria | Pseudomonadales | Pseudomonadaceae | *Pseudomonas* | Unassigned |
| *R. minimus* | ASV28 | Proteobacteria | Gammaproteobacteria | Xanthomonadales | Xanthomonadaceae | *Stenotrophomonas* | rhizophila |
| *R. minimus* | ASV37 | Proteobacteria | Gammaproteobacteria | Pseudomonadales | Pseudomonadaceae | *Pseudomonas* | Unassigned |
| *R. minimus* | ASV48 | Proteobacteria | Gammaproteobacteria | Pseudomonadales | Pseudomonadaceae | *Pseudomonas* | Unassigned |
| *R. minimus* | ASV52 | Proteobacteria | Gammaproteobacteria | Enterobacterales | Unassinged | Unassigned | Unassigned |
| *R. minimus* | ASV57 | Bacteroidota | Bacteroidia | Flavobacteriales | Weeksellaceae | *Chryseobacterium* | piscium |
| *R. minimus* | ASV67 | Actinobacteriota | Actinobacteria | Micrococcales | Microbacteriaceae | *Microbacterium* | Unassigned |
| *R. minimus* | ASV76 | Proteobacteria | Gammaproteobacteria | Burkholderiales | Oxalobacteraceae | *Duganella* | Unassigned |
| *R. minimus* | ASV77 | Proteobacteria | Gammaproteobacteria | Enterobacterales | Yersiniaceae | Unassigned | Unassigned |
| *R. minimus* | ASV87 | Proteobacteria | Gammaproteobacteria | Enterobacterales | Unassinged | Unassigned | Unassigned |
| *R. minimus* | ASV88 | Proteobacteria | Gammaproteobacteria | Aeromonadales | Aeromonadaceae | *Aeromonas* | Unassigned |
| *R. minimus* | ASV98 | Proteobacteria | Gammaproteobacteria | Pseudomonadales | Moraxellaceae | *Acinetobacter* | Unassigned |
| *R. minimus* | ASV103 | Proteobacteria | Gammaproteobacteria | Burkholderiales | Comamonadaceae | *Delftia* | Unassigned |
| *R. minimus* | ASV105 | Proteobacteria | Gammaproteobacteria | Pseudomonadales | Pseudomonadaceae | *Pseudomonas* | Unassigned |
| *R. minimus* | ASV108 | Proteobacteria | Gammaproteobacteria | Burkholderiales | Alcaligenaceae | *Alcaligenes* | Unassigned |
| *R. minimus* | ASV114 | Proteobacteria | Gammaproteobacteria | Pseudomonadales | Pseudomonadaceae | *Pseudomonas* | Unassigned |
| *R. minimus* | ASV116 | Proteobacteria | Gammaproteobacteria | Pseudomonadales | Pseudomonadaceae | *Pseudomonas* | Unassigned |
| *R. minimus* | ASV130 | Proteobacteria | Gammaproteobacteria | Pseudomonadales | Moraxellaceae | *Acinetobacter* | Unassigned |
| *R. minimus* | ASV145 | Bacteroidota | Bacteroidia | Flavobacteriales | Weeksellaceae | *Chryseobacterium* | Unassigned |
| *R. minimus* | ASV149 | Proteobacteria | Gammaproteobacteria | Burkholderiales | Oxalobacteraceae | *Massilia* | Unassigned |
| *R. minimus* | ASV152 | Actinobacteriota | Actinobacteria | Micrococcales | Micrococcaceae | Unassigned | Unassigned |
| *R. minimus* | ASV166 | Proteobacteria | Gammaproteobacteria | Burkholderiales | Comamonadaceae | Unassigned | Unassigned |
| *R. minimus* | ASV186 | Proteobacteria | Gammaproteobacteria | Burkholderiales | Comamonadaceae | Unassigned | Unassigned |
| *R. minimus* | ASV197 | Bacteroidota | Bacteroidia | Sphingobacteriales | Sphingobacteriaceae | *Pedobacter* | Unassigned |
| *R. minimus* | ASV202 | Proteobacteria | Gammaproteobacteria | Pseudomonadales | Pseudomonadaceae | *Pseudomonas* | Unassigned |
| *R. minimus* | ASV209 | Proteobacteria | Gammaproteobacteria | Xanthomonadales | Rhodanobacteraceae | *Luteibacter* | Unassigned |
| *R. minimus* | ASV215 | Proteobacteria | Alphaproteobacteria | Rhizobiales | Rhizobiaceae | *Allorhizobium-Neorhizobium-Pararhizobium-Rhizobium* | Unassigned |
| *R. minimus* | ASV255 | Proteobacteria | Gammaproteobacteria | Pseudomonadales | Pseudomonadaceae | *Pseudomonas* | Unassigned |
| *R. minimus* | ASV267 | Actinobacteriota | Actinobacteria | Micrococcales | Micrococcaceae | *Micrococcus* | Unassigned |
| *R. minimus* | ASV274 | Proteobacteria | Gammaproteobacteria | Enterobacterales | Enterobacteriaceae | Unassigned | Unassigned |
| *R. minimus* | ASV373 | Proteobacteria | Gammaproteobacteria | Pseudomonadales | Moraxellaceae | *Acinetobacter* | Unassigned |
| *R. minimus* | ASV411 | Proteobacteria | Alphaproteobacteria | Caulobacterales | Caulobacteraceae | *Brevundimonas* | Unassigned |
| *R. minimus* | ASV448 | Proteobacteria | Gammaproteobacteria | Pseudomonadales | Pseudomonadaceae | *Pseudomonas* | Unassigned |
| *R. minimus* | ASV517 | Proteobacteria | Gammaproteobacteria | Enterobacterales | Unassinged | Unassigned | Unassigned |
| *R. minimus* | ASV533 | Proteobacteria | Gammaproteobacteria | Xanthomonadales | Xanthomonadaceae | *Stenotrophomonas* | Unassigned |
| *R. minimus* | ASV561 | Proteobacteria | Gammaproteobacteria | Burkholderiales | Oxalobacteraceae | *Massilia* | Unassigned |
| *R. minimus* | ASV571 | Bacteroidota | Bacteroidia | Flavobacteriales | Weeksellaceae | *Chryseobacterium* | Unassigned |
| *R. minimus* | ASV590 | Proteobacteria | Gammaproteobacteria | Xanthomonadales | Xanthomonadaceae | *Stenotrophomonas* | Unassigned |
| *R. minimus* | ASV591 | Actinobacteriota | Actinobacteria | Micrococcales | Microbacteriaceae | *Curtobacterium* | Unassigned |
| *R. minimus* | ASV629 | Firmicutes | Bacilli | Exiguobacterales | Exiguobacteraceae | *Exiguobacterium* | Unassigned |
| *R. minimus* | ASV660 | Proteobacteria | Gammaproteobacteria | Pseudomonadales | Moraxellaceae | *Acinetobacter* | Unassigned |
| *R. minimus* | ASV707 | Proteobacteria | Gammaproteobacteria | Burkholderiales | Oxalobacteraceae | Unassigned | Unassigned |
| *R. minimus* | ASV717 | Proteobacteria | Gammaproteobacteria | Xanthomonadales | Xanthomonadaceae | *Stenotrophomonas* | Unassigned |
| *R. minimus* | ASV721 | Proteobacteria | Gammaproteobacteria | Enterobacterales | Enterobacteriaceae | Unassigned | Unassigned |
| *R. minimus* | ASV732 | Proteobacteria | Gammaproteobacteria | Xanthomonadales | Xanthomonadaceae | *Stenotrophomonas* | Unassigned |
| *R. minimus* | ASV768 | Proteobacteria | Gammaproteobacteria | Burkholderiales | Burkholderiaceae | *Burkholderia-Caballeronia-Paraburkholderia* | Unassigned |
| *R. minimus* | ASV810 | Bacteroidota | Bacteroidia | Flavobacteriales | Weeksellaceae | *Chryseobacterium* | Unassigned |
| *R. minimus* | ASV819 | Proteobacteria | Alphaproteobacteria | Rhizobiales | Beijerinckiaceae | *Bosea* | Unassigned |
| *R. minimus* | ASV926 | Proteobacteria | Alphaproteobacteria | Rhizobiales | Rhizobiaceae | *Allorhizobium-Neorhizobium-Pararhizobium-Rhizobium* | Unassigned |
| *R. minimus* | ASV957 | Proteobacteria | Gammaproteobacteria | Burkholderiales | Comamonadaceae | Unassigned | Unassigned |
| *R. minimus* | ASV987 | Proteobacteria | Gammaproteobacteria | Burkholderiales | Comamonadaceae | Unassigned | Unassigned |
| *R. minimus* | ASV988 | Firmicutes | Bacilli | Bacillales | Bacillaceae | *Bacillus* | Unassigned |
| *R. minimus* | ASV1022 | Proteobacteria | Gammaproteobacteria | Xanthomonadales | Xanthomonadaceae | *Stenotrophomonas* | Unassigned |
| *R. minimus* | ASV1023 | Bacteroidota | Bacteroidia | Flavobacteriales | Weeksellaceae | *Chryseobacterium* | Unassigned |
| *R. minimus* | ASV1093 | Proteobacteria | Gammaproteobacteria | Pseudomonadales | Pseudomonadaceae | *Pseudomonas* | Unassigned |
| *R. minimus* | ASV1163 | Proteobacteria | Alphaproteobacteria | Rhizobiales | Rhizobiaceae | *Allorhizobium-Neorhizobium-Pararhizobium-Rhizobium* | Unassigned |
| *R. minimus* | ASV1166 | Proteobacteria | Gammaproteobacteria | Xanthomonadales | Xanthomonadaceae | *Stenotrophomonas* | Unassigned |
| *R. minimus* | ASV1308 | Bacteroidota | Bacteroidia | Flavobacteriales | Flavobacteriaceae | *Flavobacterium* | Unassigned |
| *R. minimus* | ASV1313 | Firmicutes | Bacilli | Staphylococcales | Staphylococcaceae | *Staphylococcus* | Unassigned |
| *R. minimus* | ASV1412 | Bacteroidota | Bacteroidia | Flavobacteriales | Flavobacteriaceae | *Flavobacterium* | Unassigned |
| *R. minimus* | ASV1440 | Proteobacteria | Gammaproteobacteria | Burkholderiales | Comamonadaceae | *Comamonas* | Unassigned |
| *R. minimus* | ASV1457 | Proteobacteria | Gammaproteobacteria | Pseudomonadales | Moraxellaceae | *Acinetobacter* | Unassigned |
| *R. minimus* | ASV1526 | Proteobacteria | Gammaproteobacteria | Pseudomonadales | Pseudomonadaceae | *Pseudomonas* | Unassigned |
| *R. minimus* | ASV1639 | Proteobacteria | Gammaproteobacteria | Burkholderiales | Oxalobacteraceae | *Massilia* | Unassigned |
| *R. minimus* | ASV1735 | Proteobacteria | Gammaproteobacteria | Pseudomonadales | Pseudomonadaceae | *Pseudomonas* | Unassigned |
| *R. minimus* | ASV1807 | Bacteroidota | Bacteroidia | Sphingobacteriales | Sphingobacteriaceae | *Sphingobacterium* | Unassigned |
| *R. minimus* | ASV1832 | Proteobacteria | Gammaproteobacteria | Xanthomonadales | Xanthomonadaceae | *Stenotrophomonas* | Unassigned |
| *R. minimus* | ASV2157 | Proteobacteria | Gammaproteobacteria | Burkholderiales | Chitinibacteraceae | *Iodobacter* | Unassigned |
| *R. minimus* | ASV2459 | Actinobacteriota | Actinobacteria | Corynebacteriales | Nocardiaceae | *Rhodococcus* | Unassigned |
| *R. minimus* | ASV2517 | Proteobacteria | Gammaproteobacteria | Pseudomonadales | Pseudomonadaceae | *Pseudomonas* | Unassigned |
| *R. minimus* | ASV2538 | Proteobacteria | Alphaproteobacteria | Sphingomonadales | Sphingomonadaceae | *Sphingomonas* | Unassigned |
| *R. minimus* | ASV3177 | Actinobacteriota | Actinobacteria | Streptomycetales | Streptomycetaceae | *Kitasatospora* | Unassigned |
| *R. minimus* | ASV4637 | Actinobacteriota | Actinobacteria | Micrococcales | Micrococcaceae | *Paenarthrobacter* | Unassigned |
| *R. minimus* | ASV5068 | Proteobacteria | Gammaproteobacteria | Xanthomonadales | Xanthomonadaceae | *Stenotrophomonas* | Unassigned |
| *R. minimus* | ASV5454 | Firmicutes | Bacilli | Staphylococcales | Staphylococcaceae | *Staphylococcus* | Unassigned |
| *R. minimus* | ASV6197 | Actinobacteriota | Actinobacteria | Micrococcales | Brevibacteriaceae | *Brevibacterium* | Unassigned |
| *R. minimus* | ASV6722 | Bacteroidota | Bacteroidia | Flavobacteriales | Weeksellaceae | *Empedobacter* | brevis |
| *R. minimus* | ASV9383 | Firmicutes | Bacilli | Paenibacillales | Paenibacillaceae | *Paenibacillus* | Unassigned |
| *R. minimus* | ASV9995 | Bacteroidota | Bacteroidia | Sphingobacteriales | Sphingobacteriaceae | *Pedobacter* | himalayensis |
| *R. minimus* | ASV14752 | Planctomycetota | Planctomycetes | Pirellulales | Pirellulaceae | *Blastopirellula* | Unassigned |
| *L. liui* | ASV1 | Proteobacteria | Gammaproteobacteria | Pseudomonadales | Pseudomonadaceae | *Pseudomonas* | Unassigned |
| *L. liui* | ASV3 | Proteobacteria | Gammaproteobacteria | Enterobacterales | Erwiniaceae | Unassigned | Unassigned |
| *L. liui* | ASV4 | Proteobacteria | Gammaproteobacteria | Enterobacterales | Enterobacteriaceae | Unassigned | Unassigned |
| *L. liui* | ASV6 | Proteobacteria | Gammaproteobacteria | Enterobacterales | Enterobacteriaceae | Unassigned | Unassigned |
| *L. liui* | ASV8 | Proteobacteria | Gammaproteobacteria | Pseudomonadales | Pseudomonadaceae | *Pseudomonas* | Unassigned |
| *L. liui* | ASV9 | Proteobacteria | Gammaproteobacteria | Burkholderiales | Oxalobacteraceae | *Janthinobacterium* | Unassigned |
| *L. liui* | ASV10 | Proteobacteria | Gammaproteobacteria | Pseudomonadales | Pseudomonadaceae | *Pseudomonas* | Unassigned |
| *L. liui* | ASV14 | Proteobacteria | Gammaproteobacteria | Pseudomonadales | Pseudomonadaceae | *Pseudomonas* | Unassigned |
| *L. liui* | ASV20 | Proteobacteria | Gammaproteobacteria | Enterobacterales | Hafniaceae | *Hafnia-Obesumbacterium* | Unassigned |
| *L. liui* | ASV23 | Proteobacteria | Gammaproteobacteria | Pseudomonadales | Pseudomonadaceae | *Pseudomonas* | Unassigned |
| *L. liui* | ASV28 | Proteobacteria | Gammaproteobacteria | Xanthomonadales | Xanthomonadaceae | *Stenotrophomonas* | rhizophila |
| *L. liui* | ASV37 | Proteobacteria | Gammaproteobacteria | Pseudomonadales | Pseudomonadaceae | *Pseudomonas* | Unassigned |
| *L. liui* | ASV48 | Proteobacteria | Gammaproteobacteria | Pseudomonadales | Pseudomonadaceae | *Pseudomonas* | Unassigned |
| *L. liui* | ASV52 | Proteobacteria | Gammaproteobacteria | Enterobacterales | Unassinged | Unassigned | Unassigned |
| *L. liui* | ASV57 | Bacteroidota | Bacteroidia | Flavobacteriales | Weeksellaceae | *Chryseobacterium* | piscium |
| *L. liui* | ASV67 | Actinobacteriota | Actinobacteria | Micrococcales | Microbacteriaceae | *Microbacterium* | Unassigned |
| *L. liui* | ASV76 | Proteobacteria | Gammaproteobacteria | Burkholderiales | Oxalobacteraceae | *Duganella* | Unassigned |
| *L. liui* | ASV77 | Proteobacteria | Gammaproteobacteria | Enterobacterales | Yersiniaceae | Unassigned | Unassigned |
| *L. liui* | ASV87 | Proteobacteria | Gammaproteobacteria | Enterobacterales | Unassinged | Unassigned | Unassigned |
| *L. liui* | ASV88 | Proteobacteria | Gammaproteobacteria | Aeromonadales | Aeromonadaceae | *Aeromonas* | Unassigned |
| *L. liui* | ASV98 | Proteobacteria | Gammaproteobacteria | Pseudomonadales | Moraxellaceae | *Acinetobacter* | Unassigned |
| *L. liui* | ASV103 | Proteobacteria | Gammaproteobacteria | Burkholderiales | Comamonadaceae | *Delftia* | Unassigned |
| *L. liui* | ASV105 | Proteobacteria | Gammaproteobacteria | Pseudomonadales | Pseudomonadaceae | *Pseudomonas* | Unassigned |
| *L. liui* | ASV108 | Proteobacteria | Gammaproteobacteria | Burkholderiales | Alcaligenaceae | *Alcaligenes* | Unassigned |
| *L. liui* | ASV114 | Proteobacteria | Gammaproteobacteria | Pseudomonadales | Pseudomonadaceae | *Pseudomonas* | Unassigned |
| *L. liui* | ASV116 | Proteobacteria | Gammaproteobacteria | Pseudomonadales | Pseudomonadaceae | *Pseudomonas* | Unassigned |
| *L. liui* | ASV130 | Proteobacteria | Gammaproteobacteria | Pseudomonadales | Moraxellaceae | *Acinetobacter* | Unassigned |
| *L. liui* | ASV145 | Bacteroidota | Bacteroidia | Flavobacteriales | Weeksellaceae | *Chryseobacterium* | Unassigned |
| *L. liui* | ASV149 | Proteobacteria | Gammaproteobacteria | Burkholderiales | Oxalobacteraceae | *Massilia* | Unassigned |
| *L. liui* | ASV152 | Actinobacteriota | Actinobacteria | Micrococcales | Micrococcaceae | Unassigned | Unassigned |
| *L. liui* | ASV166 | Proteobacteria | Gammaproteobacteria | Burkholderiales | Comamonadaceae | Unassigned | Unassigned |
| *L. liui* | ASV186 | Proteobacteria | Gammaproteobacteria | Burkholderiales | Comamonadaceae | Unassigned | Unassigned |
| *L. liui* | ASV197 | Bacteroidota | Bacteroidia | Sphingobacteriales | Sphingobacteriaceae | *Pedobacter* | Unassigned |
| *L. liui* | ASV202 | Proteobacteria | Gammaproteobacteria | Pseudomonadales | Pseudomonadaceae | *Pseudomonas* | Unassigned |
| *L. liui* | ASV209 | Proteobacteria | Gammaproteobacteria | Xanthomonadales | Rhodanobacteraceae | *Luteibacter* | Unassigned |
| *L. liui* | ASV215 | Proteobacteria | Alphaproteobacteria | Rhizobiales | Rhizobiaceae | *Allorhizobium-Neorhizobium-Pararhizobium-Rhizobium* | Unassigned |
| *L. liui* | ASV255 | Proteobacteria | Gammaproteobacteria | Pseudomonadales | Pseudomonadaceae | *Pseudomonas* | Unassigned |
| *L. liui* | ASV267 | Actinobacteriota | Actinobacteria | Micrococcales | Micrococcaceae | *Micrococcus* | Unassigned |
| *L. liui* | ASV274 | Proteobacteria | Gammaproteobacteria | Enterobacterales | Enterobacteriaceae | Unassigned | Unassigned |
| *L. liui* | ASV373 | Proteobacteria | Gammaproteobacteria | Pseudomonadales | Moraxellaceae | *Acinetobacter* | Unassigned |
| *L. liui* | ASV411 | Proteobacteria | Alphaproteobacteria | Caulobacterales | Caulobacteraceae | *Brevundimonas* | Unassigned |
| *L. liui* | ASV448 | Proteobacteria | Gammaproteobacteria | Pseudomonadales | Pseudomonadaceae | *Pseudomonas* | Unassigned |
| *L. liui* | ASV517 | Proteobacteria | Gammaproteobacteria | Enterobacterales | Unassinged | Unassigned | Unassigned |
| *L. liui* | ASV533 | Proteobacteria | Gammaproteobacteria | Xanthomonadales | Xanthomonadaceae | *Stenotrophomonas* | Unassigned |
| *L. liui* | ASV561 | Proteobacteria | Gammaproteobacteria | Burkholderiales | Oxalobacteraceae | *Massilia* | Unassigned |
| *L. liui* | ASV571 | Bacteroidota | Bacteroidia | Flavobacteriales | Weeksellaceae | *Chryseobacterium* | Unassigned |
| *L. liui* | ASV590 | Proteobacteria | Gammaproteobacteria | Xanthomonadales | Xanthomonadaceae | *Stenotrophomonas* | Unassigned |
| *L. liui* | ASV591 | Actinobacteriota | Actinobacteria | Micrococcales | Microbacteriaceae | *Curtobacterium* | Unassigned |
| *L. liui* | ASV629 | Firmicutes | Bacilli | Exiguobacterales | Exiguobacteraceae | *Exiguobacterium* | Unassigned |
| *L. liui* | ASV660 | Proteobacteria | Gammaproteobacteria | Pseudomonadales | Moraxellaceae | *Acinetobacter* | Unassigned |
| *L. liui* | ASV707 | Proteobacteria | Gammaproteobacteria | Burkholderiales | Oxalobacteraceae | Unassigned | Unassigned |
| *L. liui* | ASV717 | Proteobacteria | Gammaproteobacteria | Xanthomonadales | Xanthomonadaceae | *Stenotrophomonas* | Unassigned |
| *L. liui* | ASV721 | Proteobacteria | Gammaproteobacteria | Enterobacterales | Enterobacteriaceae | Unassigned | Unassigned |
| *L. liui* | ASV732 | Proteobacteria | Gammaproteobacteria | Xanthomonadales | Xanthomonadaceae | *Stenotrophomonas* | Unassigned |
| *L. liui* | ASV768 | Proteobacteria | Gammaproteobacteria | Burkholderiales | Burkholderiaceae | *Burkholderia-Caballeronia-Paraburkholderia* | Unassigned |
| *L. liui* | ASV810 | Bacteroidota | Bacteroidia | Flavobacteriales | Weeksellaceae | *Chryseobacterium* | Unassigned |
| *L. liui* | ASV819 | Proteobacteria | Alphaproteobacteria | Rhizobiales | Beijerinckiaceae | *Bosea* | Unassigned |
| *L. liui* | ASV904 | Proteobacteria | Gammaproteobacteria | Burkholderiales | Oxalobacteraceae | *Duganella* | Unassigned |
| *L. liui* | ASV926 | Proteobacteria | Alphaproteobacteria | Rhizobiales | Rhizobiaceae | *Allorhizobium-Neorhizobium-Pararhizobium-Rhizobium* | Unassigned |
| *L. liui* | ASV957 | Proteobacteria | Gammaproteobacteria | Burkholderiales | Comamonadaceae | Unassigned | Unassigned |
| *L. liui* | ASV987 | Proteobacteria | Gammaproteobacteria | Burkholderiales | Comamonadaceae | Unassigned | Unassigned |
| *L. liui* | ASV988 | Firmicutes | Bacilli | Bacillales | Bacillaceae | *Bacillus* | Unassigned |
| *L. liui* | ASV1004 | Proteobacteria | Gammaproteobacteria | Pseudomonadales | Pseudomonadaceae | *Pseudomonas* | Unassigned |
| *L. liui* | ASV1022 | Proteobacteria | Gammaproteobacteria | Xanthomonadales | Xanthomonadaceae | *Stenotrophomonas* | Unassigned |
| *L. liui* | ASV1163 | Proteobacteria | Alphaproteobacteria | Rhizobiales | Rhizobiaceae | *Allorhizobium-Neorhizobium-Pararhizobium-Rhizobium* | Unassigned |
| *L. liui* | ASV1166 | Proteobacteria | Gammaproteobacteria | Xanthomonadales | Xanthomonadaceae | *Stenotrophomonas* | Unassigned |
| *L. liui* | ASV1308 | Bacteroidota | Bacteroidia | Flavobacteriales | Flavobacteriaceae | *Flavobacterium* | Unassigned |
| *L. liui* | ASV1412 | Bacteroidota | Bacteroidia | Flavobacteriales | Flavobacteriaceae | *Flavobacterium* | Unassigned |
| *L. liui* | ASV1440 | Proteobacteria | Gammaproteobacteria | Burkholderiales | Comamonadaceae | *Comamonas* | Unassigned |
| *L. liui* | ASV1457 | Proteobacteria | Gammaproteobacteria | Pseudomonadales | Moraxellaceae | *Acinetobacter* | Unassigned |
| *L. liui* | ASV1526 | Proteobacteria | Gammaproteobacteria | Pseudomonadales | Pseudomonadaceae | *Pseudomonas* | Unassigned |
| *L. liui* | ASV1639 | Proteobacteria | Gammaproteobacteria | Burkholderiales | Oxalobacteraceae | *Massilia* | Unassigned |
| *L. liui* | ASV1642 | Proteobacteria | Alphaproteobacteria | Sphingomonadales | Sphingomonadaceae | *Novosphingobium* | Unassigned |
| *L. liui* | ASV1735 | Proteobacteria | Gammaproteobacteria | Pseudomonadales | Pseudomonadaceae | *Pseudomonas* | Unassigned |
| *L. liui* | ASV1807 | Bacteroidota | Bacteroidia | Sphingobacteriales | Sphingobacteriaceae | *Sphingobacterium* | Unassigned |
| *L. liui* | ASV1832 | Proteobacteria | Gammaproteobacteria | Xanthomonadales | Xanthomonadaceae | *Stenotrophomonas* | Unassigned |
| *L. liui* | ASV2157 | Proteobacteria | Gammaproteobacteria | Burkholderiales | Chitinibacteraceae | *Iodobacter* | Unassigned |
| *L. liui* | ASV2459 | Actinobacteriota | Actinobacteria | Corynebacteriales | Nocardiaceae | *Rhodococcus* | Unassigned |
| *L. liui* | ASV2517 | Proteobacteria | Gammaproteobacteria | Pseudomonadales | Pseudomonadaceae | *Pseudomonas* | Unassigned |
| *L. liui* | ASV2538 | Proteobacteria | Alphaproteobacteria | Sphingomonadales | Sphingomonadaceae | *Sphingomonas* | Unassigned |
| *L. liui* | ASV2816 | Proteobacteria | Gammaproteobacteria | Pseudomonadales | Pseudomonadaceae | *Pseudomonas* | Unassigned |
| *L. liui* | ASV2980 | Proteobacteria | Gammaproteobacteria | Burkholderiales | Chromobacteriaceae | *Chromobacterium* | Unassigned |
| *L. liui* | ASV3254 | Actinobacteriota | Actinobacteria | Streptomycetales | Streptomycetaceae | *Streptomyces* | Unassigned |
| *L. liui* | ASV3901 | Proteobacteria | Gammaproteobacteria | Pseudomonadales | Pseudomonadaceae | *Pseudomonas* | Unassigned |
| *L. liui* | ASV4379 | Proteobacteria | Gammaproteobacteria | Burkholderiales | Burkholderiaceae | *Burkholderia-Caballeronia-Paraburkholderia* | Unassigned |
| *L. liui* | ASV4637 | Actinobacteriota | Actinobacteria | Micrococcales | Micrococcaceae | *Paenarthrobacter* | Unassigned |
| *L. liui* | ASV4786 | Bacteroidota | Bacteroidia | Sphingobacteriales | Sphingobacteriaceae | *Pedobacter* | Unassigned |
| *L. liui* | ASV6197 | Actinobacteriota | Actinobacteria | Micrococcales | Brevibacteriaceae | *Brevibacterium* | Unassigned |
| *L. liui* | ASV6951 | Proteobacteria | Gammaproteobacteria | Xanthomonadales | Xanthomonadaceae | Unassigned | Unassigned |
| *L. liui* | ASV7678 | Proteobacteria | Alphaproteobacteria | Caulobacterales | Caulobacteraceae | *Brevundimonas* | terrae |
| *L. liui* | ASV7955 | Firmicutes | Bacilli | Bacillales | Bacillaceae | *Bacillus* | Unassigned |
| *L. liui* | ASV8564 | Actinobacteriota | Actinobacteria | Micrococcales | Microbacteriaceae | *Microbacterium* | Unassigned |
| *L. liui* | ASV12717 | Bacteroidota | Bacteroidia | Sphingobacteriales | Sphingobacteriaceae | *Pedobacter* | Unassigned |
| *L. liui* | ASV14752 | Planctomycetota | Planctomycetes | Pirellulales | Pirellulaceae | *Blastopirellula* | Unassigned |
| *L. liui* | ASV21601 | Bacteroidota | Bacteroidia | Flavobacteriales | Flavobacteriaceae | *Flavobacterium* | Unassigned |
| *L. liui* | ASV25184 | Bacteroidota | Bacteroidia | Unassigned | Unassinged | Unassigned | Unassigned |
| *A. chunganensis* | ASV1 | Proteobacteria | Gammaproteobacteria | Pseudomonadales | Pseudomonadaceae | *Pseudomonas* | Unassigned |
| *A. chunganensis* | ASV3 | Proteobacteria | Gammaproteobacteria | Enterobacterales | Erwiniaceae | Unassigned | Unassigned |
| *A. chunganensis* | ASV4 | Proteobacteria | Gammaproteobacteria | Enterobacterales | Enterobacteriaceae | Unassigned | Unassigned |
| *A. chunganensis* | ASV6 | Proteobacteria | Gammaproteobacteria | Enterobacterales | Enterobacteriaceae | Unassigned | Unassigned |
| *A. chunganensis* | ASV8 | Proteobacteria | Gammaproteobacteria | Pseudomonadales | Pseudomonadaceae | *Pseudomonas* | Unassigned |
| *A. chunganensis* | ASV9 | Proteobacteria | Gammaproteobacteria | Burkholderiales | Oxalobacteraceae | *Janthinobacterium* | Unassigned |
| *A. chunganensis* | ASV10 | Proteobacteria | Gammaproteobacteria | Pseudomonadales | Pseudomonadaceae | *Pseudomonas* | Unassigned |
| *A. chunganensis* | ASV14 | Proteobacteria | Gammaproteobacteria | Pseudomonadales | Pseudomonadaceae | *Pseudomonas* | Unassigned |
| *A. chunganensis* | ASV20 | Proteobacteria | Gammaproteobacteria | Enterobacterales | Hafniaceae | *Hafnia-Obesumbacterium* | Unassigned |
| *A. chunganensis* | ASV23 | Proteobacteria | Gammaproteobacteria | Pseudomonadales | Pseudomonadaceae | *Pseudomonas* | Unassigned |
| *A. chunganensis* | ASV28 | Proteobacteria | Gammaproteobacteria | Xanthomonadales | Xanthomonadaceae | *Stenotrophomonas* | rhizophila |
| *A. chunganensis* | ASV37 | Proteobacteria | Gammaproteobacteria | Pseudomonadales | Pseudomonadaceae | *Pseudomonas* | Unassigned |
| *A. chunganensis* | ASV48 | Proteobacteria | Gammaproteobacteria | Pseudomonadales | Pseudomonadaceae | *Pseudomonas* | Unassigned |
| *A. chunganensis* | ASV52 | Proteobacteria | Gammaproteobacteria | Enterobacterales | Unassinged | Unassigned | Unassigned |
| *A. chunganensis* | ASV57 | Bacteroidota | Bacteroidia | Flavobacteriales | Weeksellaceae | *Chryseobacterium* | piscium |
| *A. chunganensis* | ASV67 | Actinobacteriota | Actinobacteria | Micrococcales | Microbacteriaceae | *Microbacterium* | Unassigned |
| *A. chunganensis* | ASV76 | Proteobacteria | Gammaproteobacteria | Burkholderiales | Oxalobacteraceae | *Duganella* | Unassigned |
| *A. chunganensis* | ASV77 | Proteobacteria | Gammaproteobacteria | Enterobacterales | Yersiniaceae | Unassigned | Unassigned |
| *A. chunganensis* | ASV87 | Proteobacteria | Gammaproteobacteria | Enterobacterales | Unassinged | Unassigned | Unassigned |
| *A. chunganensis* | ASV88 | Proteobacteria | Gammaproteobacteria | Aeromonadales | Aeromonadaceae | *Aeromonas* | Unassigned |
| *A. chunganensis* | ASV98 | Proteobacteria | Gammaproteobacteria | Pseudomonadales | Moraxellaceae | *Acinetobacter* | Unassigned |
| *A. chunganensis* | ASV103 | Proteobacteria | Gammaproteobacteria | Burkholderiales | Comamonadaceae | *Delftia* | Unassigned |
| *A. chunganensis* | ASV105 | Proteobacteria | Gammaproteobacteria | Pseudomonadales | Pseudomonadaceae | *Pseudomonas* | Unassigned |
| *A. chunganensis* | ASV108 | Proteobacteria | Gammaproteobacteria | Burkholderiales | Alcaligenaceae | *Alcaligenes* | Unassigned |
| *A. chunganensis* | ASV114 | Proteobacteria | Gammaproteobacteria | Pseudomonadales | Pseudomonadaceae | *Pseudomonas* | Unassigned |
| *A. chunganensis* | ASV116 | Proteobacteria | Gammaproteobacteria | Pseudomonadales | Pseudomonadaceae | *Pseudomonas* | Unassigned |
| *A. chunganensis* | ASV130 | Proteobacteria | Gammaproteobacteria | Pseudomonadales | Moraxellaceae | *Acinetobacter* | Unassigned |
| *A. chunganensis* | ASV145 | Bacteroidota | Bacteroidia | Flavobacteriales | Weeksellaceae | *Chryseobacterium* | Unassigned |
| *A. chunganensis* | ASV149 | Proteobacteria | Gammaproteobacteria | Burkholderiales | Oxalobacteraceae | *Massilia* | Unassigned |
| *A. chunganensis* | ASV152 | Actinobacteriota | Actinobacteria | Micrococcales | Micrococcaceae | Unassigned | Unassigned |
| *A. chunganensis* | ASV166 | Proteobacteria | Gammaproteobacteria | Burkholderiales | Comamonadaceae | Unassigned | Unassigned |
| *A. chunganensis* | ASV186 | Proteobacteria | Gammaproteobacteria | Burkholderiales | Comamonadaceae | Unassigned | Unassigned |
| *A. chunganensis* | ASV197 | Bacteroidota | Bacteroidia | Sphingobacteriales | Sphingobacteriaceae | *Pedobacter* | Unassigned |
| *A. chunganensis* | ASV202 | Proteobacteria | Gammaproteobacteria | Pseudomonadales | Pseudomonadaceae | *Pseudomonas* | Unassigned |
| *A. chunganensis* | ASV209 | Proteobacteria | Gammaproteobacteria | Xanthomonadales | Rhodanobacteraceae | *Luteibacter* | Unassigned |
| *A. chunganensis* | ASV215 | Proteobacteria | Alphaproteobacteria | Rhizobiales | Rhizobiaceae | *Allorhizobium-Neorhizobium-Pararhizobium-Rhizobium* | Unassigned |
| *A. chunganensis* | ASV255 | Proteobacteria | Gammaproteobacteria | Pseudomonadales | Pseudomonadaceae | *Pseudomonas* | Unassigned |
| *A. chunganensis* | ASV267 | Actinobacteriota | Actinobacteria | Micrococcales | Micrococcaceae | *Micrococcus* | Unassigned |
| *A. chunganensis* | ASV274 | Proteobacteria | Gammaproteobacteria | Enterobacterales | Enterobacteriaceae | Unassigned | Unassigned |
| *A. chunganensis* | ASV373 | Proteobacteria | Gammaproteobacteria | Pseudomonadales | Moraxellaceae | *Acinetobacter* | Unassigned |
| *A. chunganensis* | ASV411 | Proteobacteria | Alphaproteobacteria | Caulobacterales | Caulobacteraceae | *Brevundimonas* | Unassigned |
| *A. chunganensis* | ASV448 | Proteobacteria | Gammaproteobacteria | Pseudomonadales | Pseudomonadaceae | *Pseudomonas* | Unassigned |
| *A. chunganensis* | ASV517 | Proteobacteria | Gammaproteobacteria | Enterobacterales | Unassinged | Unassigned | Unassigned |
| *A. chunganensis* | ASV533 | Proteobacteria | Gammaproteobacteria | Xanthomonadales | Xanthomonadaceae | *Stenotrophomonas* | Unassigned |
| *A. chunganensis* | ASV555 | Proteobacteria | Gammaproteobacteria | Enterobacterales | Enterobacteriaceae | Unassigned | Unassigned |
| *A. chunganensis* | ASV571 | Bacteroidota | Bacteroidia | Flavobacteriales | Weeksellaceae | *Chryseobacterium* | Unassigned |
| *A. chunganensis* | ASV591 | Actinobacteriota | Actinobacteria | Micrococcales | Microbacteriaceae | *Curtobacterium* | Unassigned |
| *A. chunganensis* | ASV629 | Firmicutes | Bacilli | Exiguobacterales | Exiguobacteraceae | *Exiguobacterium* | Unassigned |
| *A. chunganensis* | ASV660 | Proteobacteria | Gammaproteobacteria | Pseudomonadales | Moraxellaceae | *Acinetobacter* | Unassigned |
| *A. chunganensis* | ASV707 | Proteobacteria | Gammaproteobacteria | Burkholderiales | Oxalobacteraceae | Unassigned | Unassigned |
| *A. chunganensis* | ASV717 | Proteobacteria | Gammaproteobacteria | Xanthomonadales | Xanthomonadaceae | *Stenotrophomonas* | Unassigned |
| *A. chunganensis* | ASV721 | Proteobacteria | Gammaproteobacteria | Enterobacterales | Enterobacteriaceae | Unassigned | Unassigned |
| *A. chunganensis* | ASV732 | Proteobacteria | Gammaproteobacteria | Xanthomonadales | Xanthomonadaceae | *Stenotrophomonas* | Unassigned |
| *A. chunganensis* | ASV768 | Proteobacteria | Gammaproteobacteria | Burkholderiales | Burkholderiaceae | *Burkholderia-Caballeronia-Paraburkholderia* | Unassigned |
| *A. chunganensis* | ASV810 | Bacteroidota | Bacteroidia | Flavobacteriales | Weeksellaceae | *Chryseobacterium* | Unassigned |
| *A. chunganensis* | ASV819 | Proteobacteria | Alphaproteobacteria | Rhizobiales | Beijerinckiaceae | *Bosea* | Unassigned |
| *A. chunganensis* | ASV904 | Proteobacteria | Gammaproteobacteria | Burkholderiales | Oxalobacteraceae | *Duganella* | Unassigned |
| *A. chunganensis* | ASV926 | Proteobacteria | Alphaproteobacteria | Rhizobiales | Rhizobiaceae | *Allorhizobium-Neorhizobium-Pararhizobium-Rhizobium* | Unassigned |
| *A. chunganensis* | ASV957 | Proteobacteria | Gammaproteobacteria | Burkholderiales | Comamonadaceae | Unassigned | Unassigned |
| *A. chunganensis* | ASV987 | Proteobacteria | Gammaproteobacteria | Burkholderiales | Comamonadaceae | Unassigned | Unassigned |
| *A. chunganensis* | ASV988 | Firmicutes | Bacilli | Bacillales | Bacillaceae | *Bacillus* | Unassigned |
| *A. chunganensis* | ASV1004 | Proteobacteria | Gammaproteobacteria | Pseudomonadales | Pseudomonadaceae | *Pseudomonas* | Unassigned |
| *A. chunganensis* | ASV1022 | Proteobacteria | Gammaproteobacteria | Xanthomonadales | Xanthomonadaceae | *Stenotrophomonas* | Unassigned |
| *A. chunganensis* | ASV1023 | Bacteroidota | Bacteroidia | Flavobacteriales | Weeksellaceae | *Chryseobacterium* | Unassigned |
| *A. chunganensis* | ASV1093 | Proteobacteria | Gammaproteobacteria | Pseudomonadales | Pseudomonadaceae | *Pseudomonas* | Unassigned |
| *A. chunganensis* | ASV1163 | Proteobacteria | Alphaproteobacteria | Rhizobiales | Rhizobiaceae | *Allorhizobium-Neorhizobium-Pararhizobium-Rhizobium* | Unassigned |
| *A. chunganensis* | ASV1166 | Proteobacteria | Gammaproteobacteria | Xanthomonadales | Xanthomonadaceae | *Stenotrophomonas* | Unassigned |
| *A. chunganensis* | ASV1308 | Bacteroidota | Bacteroidia | Flavobacteriales | Flavobacteriaceae | *Flavobacterium* | Unassigned |
| *A. chunganensis* | ASV1412 | Bacteroidota | Bacteroidia | Flavobacteriales | Flavobacteriaceae | *Flavobacterium* | Unassigned |
| *A. chunganensis* | ASV1440 | Proteobacteria | Gammaproteobacteria | Burkholderiales | Comamonadaceae | *Comamonas* | Unassigned |
| *A. chunganensis* | ASV1457 | Proteobacteria | Gammaproteobacteria | Pseudomonadales | Moraxellaceae | *Acinetobacter* | Unassigned |
| *A. chunganensis* | ASV1526 | Proteobacteria | Gammaproteobacteria | Pseudomonadales | Pseudomonadaceae | *Pseudomonas* | Unassigned |
| *A. chunganensis* | ASV1639 | Proteobacteria | Gammaproteobacteria | Burkholderiales | Oxalobacteraceae | *Massilia* | Unassigned |
| *A. chunganensis* | ASV1642 | Proteobacteria | Alphaproteobacteria | Sphingomonadales | Sphingomonadaceae | *Novosphingobium* | Unassigned |
| *A. chunganensis* | ASV1735 | Proteobacteria | Gammaproteobacteria | Pseudomonadales | Pseudomonadaceae | *Pseudomonas* | Unassigned |
| *A. chunganensis* | ASV1807 | Bacteroidota | Bacteroidia | Sphingobacteriales | Sphingobacteriaceae | *Sphingobacterium* | Unassigned |
| *A. chunganensis* | ASV1832 | Proteobacteria | Gammaproteobacteria | Xanthomonadales | Xanthomonadaceae | *Stenotrophomonas* | Unassigned |
| *A. chunganensis* | ASV2157 | Proteobacteria | Gammaproteobacteria | Burkholderiales | Chitinibacteraceae | *Iodobacter* | Unassigned |
| *A. chunganensis* | ASV2395 | Bacteroidota | Bacteroidia | Flavobacteriales | Flavobacteriaceae | *Flavobacterium* | Unassigned |
| *A. chunganensis* | ASV2459 | Actinobacteriota | Actinobacteria | Corynebacteriales | Nocardiaceae | *Rhodococcus* | Unassigned |
| *A. chunganensis* | ASV2816 | Proteobacteria | Gammaproteobacteria | Pseudomonadales | Pseudomonadaceae | *Pseudomonas* | Unassigned |
| *A. chunganensis* | ASV2980 | Proteobacteria | Gammaproteobacteria | Burkholderiales | Chromobacteriaceae | *Chromobacterium* | Unassigned |
| *A. chunganensis* | ASV3901 | Proteobacteria | Gammaproteobacteria | Pseudomonadales | Pseudomonadaceae | *Pseudomonas* | Unassigned |
| *A. chunganensis* | ASV4168 | Bacteroidota | Bacteroidia | Flavobacteriales | Flavobacteriaceae | *Flavobacterium* | Unassigned |
| *A. chunganensis* | ASV5385 | Proteobacteria | Gammaproteobacteria | Pseudomonadales | Pseudomonadaceae | *Pseudomonas* | Unassigned |
| *A. chunganensis* | ASV6197 | Actinobacteriota | Actinobacteria | Micrococcales | Brevibacteriaceae | *Brevibacterium* | Unassigned |
| *A. chunganensis* | ASV6547 | Proteobacteria | Gammaproteobacteria | Burkholderiales | Burkholderiaceae | *Burkholderia-Caballeronia-Paraburkholderia* | Unassigned |
| *A. chunganensis* | ASV6722 | Bacteroidota | Bacteroidia | Flavobacteriales | Weeksellaceae | *Empedobacter* | brevis |
| *A. chunganensis* | ASV7678 | Proteobacteria | Alphaproteobacteria | Caulobacterales | Caulobacteraceae | *Brevundimonas* | terrae |
| *A. chunganensis* | ASV9995 | Bacteroidota | Bacteroidia | Sphingobacteriales | Sphingobacteriaceae | *Pedobacter* | himalayensis |
| *A. chunganensis* | ASV14752 | Planctomycetota | Planctomycetes | Pirellulales | Pirellulaceae | *Blastopirellula* | Unassigned |
| *A. chunganensis* | ASV16781 | Planctomycetota | Phycisphaerae | Phycisphaerales | Phycisphaeraceae | *SM1A02* | Unassigned |
| *A. chunganensis* | ASV18515 | Proteobacteria | Gammaproteobacteria | Enterobacterales | Enterobacteriaceae | Unassigned | Unassigned |
| *A. chunganensis* | ASV19026 | Proteobacteria | Gammaproteobacteria | Xanthomonadales | Rhodanobacteraceae | *Luteibacter* | anthropi |
| *A. chunganensis* | ASV20629 | Proteobacteria | Gammaproteobacteria | Burkholderiales | Chromobacteriaceae | *Chromobacterium* | Unassigned |
| *A. chunganensis* | ASV23574 | Unassigned | Unassigned | Unassigned | Unassinged | Unassigned | Unassigned |

**Table S6.** Results of the Kolmogorov-Smirnov test comparing bootstrapped node attributes of microbial networks of infected and uninfected individuals of *T. rhododiscus*.

| **Comparison** | **Degree** | **Betweenness** | **Closeness** | **Transitivity** |
| --- | --- | --- | --- | --- |
| *Bd*- vs *Bd*+ | 0.4429*** | 0.1707*** | 0.6041*** | 0.3109*** |

**Table S7.** Key topological features of micriobial co-occurrence networks.

|  | **Nodes** | **Edges** | **Positive**  **ratio** | **Average clustering  coefficient** | **Average degree** | **Average path  length** | **Diameter** | **Density** | **Modularity** | **Betweenness  centralization** | **Degree  centralization** |
| --- | --- | --- | --- | --- | --- | --- | --- | --- | --- | --- | --- |
| *Bd*- | 295 | 9031 | 0.97 | 0.868 | 61.227 | 2.971 | 11 | 0.208 | 0.138 | 0.077 | 0.261 |
| *Bd*+ | 311 | 13189 | 0.98 | 0.809 | 84.817 | 2.401 | 10 | 0.274 | 0.114 | 0.030 | 0.278 |

**Table S8.** The genera were classified as connectors in microbial community networks of uninfected frogs (*Bd-*) and infected frogs (*Bd+*). Bold fonts indicate the putative anti-*Bd* bacteria.

|  | **ASV** | **Kingdom** | **Phylum** | **Class** | **Order** | **Family** | **Genus** |
| --- | --- | --- | --- | --- | --- | --- | --- |
|  | ASV257 | Bacteria | Bacteroidota | Bacteroidia | Bacteroidales | Tannerellaceae | *Parabacteroides* |
|  | ASV111 | Bacteria | Desulfobacterota | Desulfovibrionia | Desulfovibrionales | Desulfovibrionaceae | *Bilophila* |
|  | ASV269 | Bacteria | Firmicutes | Clostridia | Lachnospirales | Lachnospiraceae | *Lachnoclostridium* |
|  | ASV42 | Bacteria | Firmicutes | Clostridia | Lachnospirales | Lachnospiraceae | Unassigned |
| *Bd-* | **ASV105** | Bacteria | Proteobacteria | Gammaproteobacteria | Pseudomonadales | Pseudomonadaceae | *Pseudomonas* |
|  | ASV337 | Bacteria | Proteobacteria | Gammaproteobacteria | UBA10353_marine_group | UBA10353_marine_group | *UBA10353_marine_group* |
|  | ASV374 | Bacteria | Verrucomicrobiota | Verrucomicrobiae | Verrucomicrobiales | Akkermansiaceae | *Akkermansia* |
|  | ASV111 | Fungi | Ascomycota | Dothideomycetes | Pleosporales | Unassigned | Unassigned |
|  | ASV62 | Fungi | Unassigned | Unassigned | Unassigned | Unassigned | Unassigned |
|  | ASV831 | Bacteria | Acidobacteriota | Holophagae | Subgroup_7 | Subgroup_7 | *Subgroup_7* |
|  | ASV569 | Bacteria | Acidobacteriota | Vicinamibacteria | Subgroup_17 | Subgroup_17 | *Subgroup_17* |
|  | **ASV152** | Bacteria | Acidobacteriota | Actinobacteria | Micrococcales | Micrococcaceae | Unassigned |
|  | ASV216 | Bacteria | Bacteroidota | Bacteroidia | Bacteroidales | Muribaculaceae | *Muribaculaceae* |
|  | ASV364 | Bacteria | Bacteroidota | Bacteroidia | Bacteroidales | Muribaculaceae | *Muribaculaceae* |
|  | ASV419 | Bacteria | Bacteroidota | Bacteroidia | Bacteroidales | Muribaculaceae | *Muribaculaceae* |
|  | ASV455 | Bacteria | Bacteroidota | Bacteroidia | Bacteroidales | Muribaculaceae | *Muribaculaceae* |
|  | ASV1080 | Bacteria | Bacteroidota | Bacteroidia | Bacteroidales | Prevotellaceae | *Alloprevotella* |
|  | ASV556 | Bacteria | Bacteroidota | Bacteroidia | Bacteroidales | Tannerellaceae | *Macellibacteroides* |
| *Bd+* | ASV82 | Bacteria | Bacteroidota | Bacteroidia | Bacteroidales | Tannerellaceae | Unassigned |
|  | ASV73 | Bacteria | Firmicutes | Bacilli | Lactobacillales | Lactobacillaceae | *Lactobacillus* |
|  | ASV21 | Bacteria | Firmicutes | Bacilli | Staphylococcales | Staphylococcaceae | *Staphylococcus* |
|  | ASV1011 | Bacteria | Firmicutes | Clostridia | Lachnospirales | Lachnospiraceae | *Lachnospiraceae_NK4A136_group* |
|  | ASV205 | Bacteria | Firmicutes | Clostridia | Oscillospirales | Ruminococcaceae | *Faecalibacterium* |
|  | ASV287 | Bacteria | Firmicutes | Clostridia | Oscillospirales | Ruminococcaceae | *Subdoligranulum* |
|  | ASV642 | Bacteria | Firmicutes | Negativicutes | Veillonellales-Selenomonadales | Selenomonadaceae | *Megamonas* |
|  | ASV84 | Bacteria | Proteobacteria | Alphaproteobacteria | Rhizobiales | Beijerinckiaceae | Unassigned |
|  | ASV458 | Bacteria | Proteobacteria | Gammaproteobacteria | Burkholderiales | Comamonadaceae | Unassigned |
|  | ASV680 | Bacteria | Proteobacteria | Gammaproteobacteria | Burkholderiales | SC-I-84 | *SC-I-84* |
|  | **ASV37** | Bacteria | Proteobacteria | Gammaproteobacteria | Pseudomonadales | Pseudomonadaceae | *Pseudomonas* |
|  | ASV22 | Fungi | Ascomycota | Dothideomycetes | Capnodiales | Unassigned | Unassigned |
|  | ASV28 | Fungi | Ascomycota | Dothideomycetes | Pleosporales | Cucurbitariaceae | *Pyrenochaeta* |
