# Supplementary material for: Associations of Batrachochytrium dendrobatidis with skin bacteria and fungi on Asian amphibian hosts
Source: ISME Commun. 2023 Nov 22;3:123. doi: 10.1038/s43705-023-00332-7 (PMC10665332; doi:10.1038/s43705-023-00332-7)
Supplement: Supplementary file 2 — Supplemental Materials Figures [file 43705_2023_332_MOESM2_ESM.docx]

**Supplementary Information – Figures**

**Associations of *Batrachochytrium dendrobatidis* with skin bacteria and fungi on Asian amphibian hosts**

Dan Sun^1^, Jayampathi Herath^1,2^, Shipeng Zhou^1^, Gajaba Ellepola^1,3^, Madhava Meegaskumbura^1,^*

^1^ Guangxi Key Laboratory for Forest Ecology and Conservation, College of Forestry, Guangxi University; Nanning, Guangxi 530000, People’s Republic of China

^2^ School of Biomedical Sciences, International Institute of Health Sciences (IIHS), No 704 Negombo Rd, Welisara 71722, Sri Lanka

^3^ Department of Zoology, Faculty of Science, University of Peradeniya, Peradeniya, KY20400, Sri Lanka

* Corresponding Author E-mail: madhava_m@mac.com


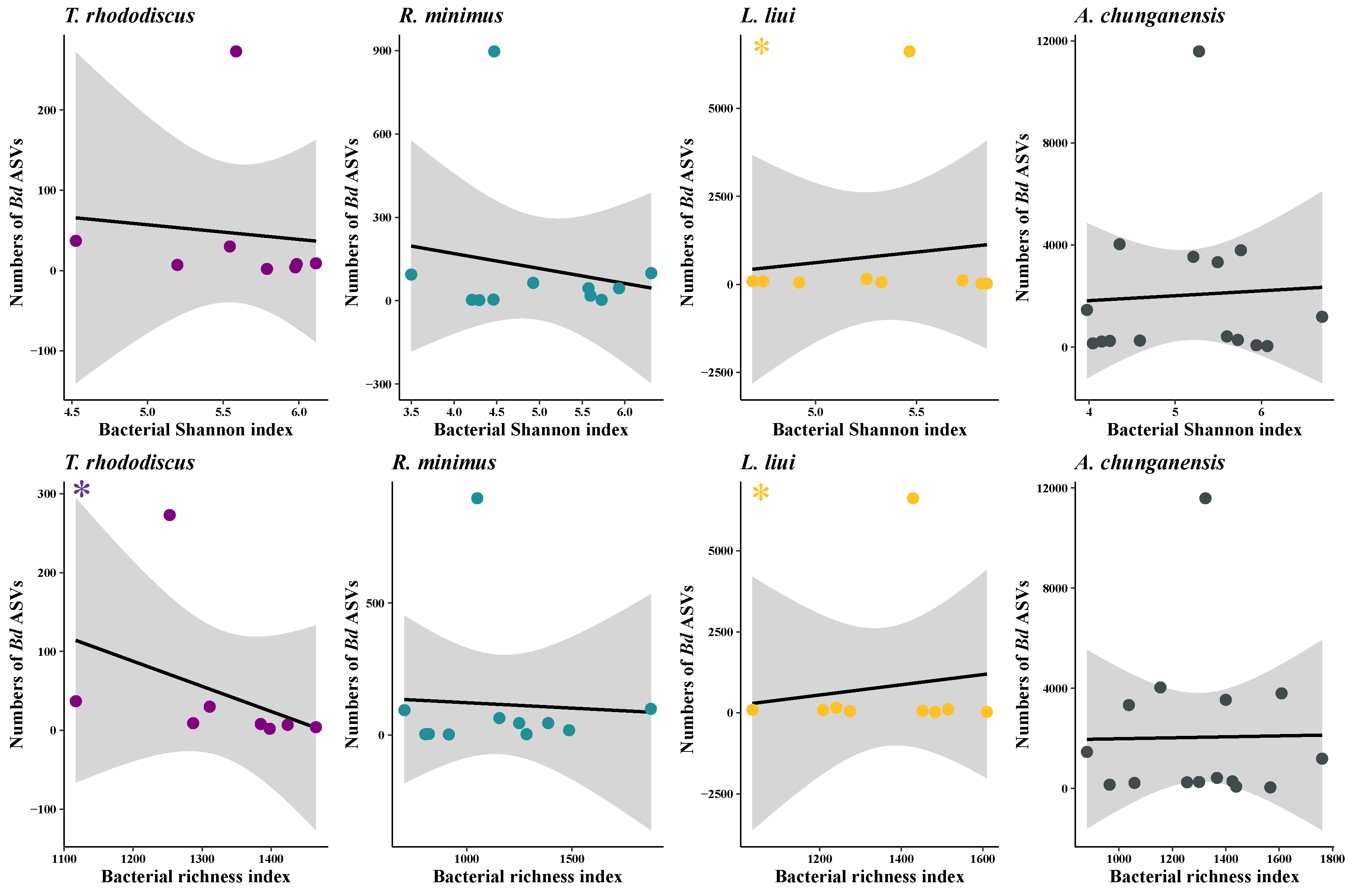


**Supplementary Figure S1**

Linear regressions of bacterial alpha diversity values and *Bd* infection intensity (numbers of *Bd* ASVs). Shading represents 95% confidence intervals for each species. Asterisks indicate significant correlations.


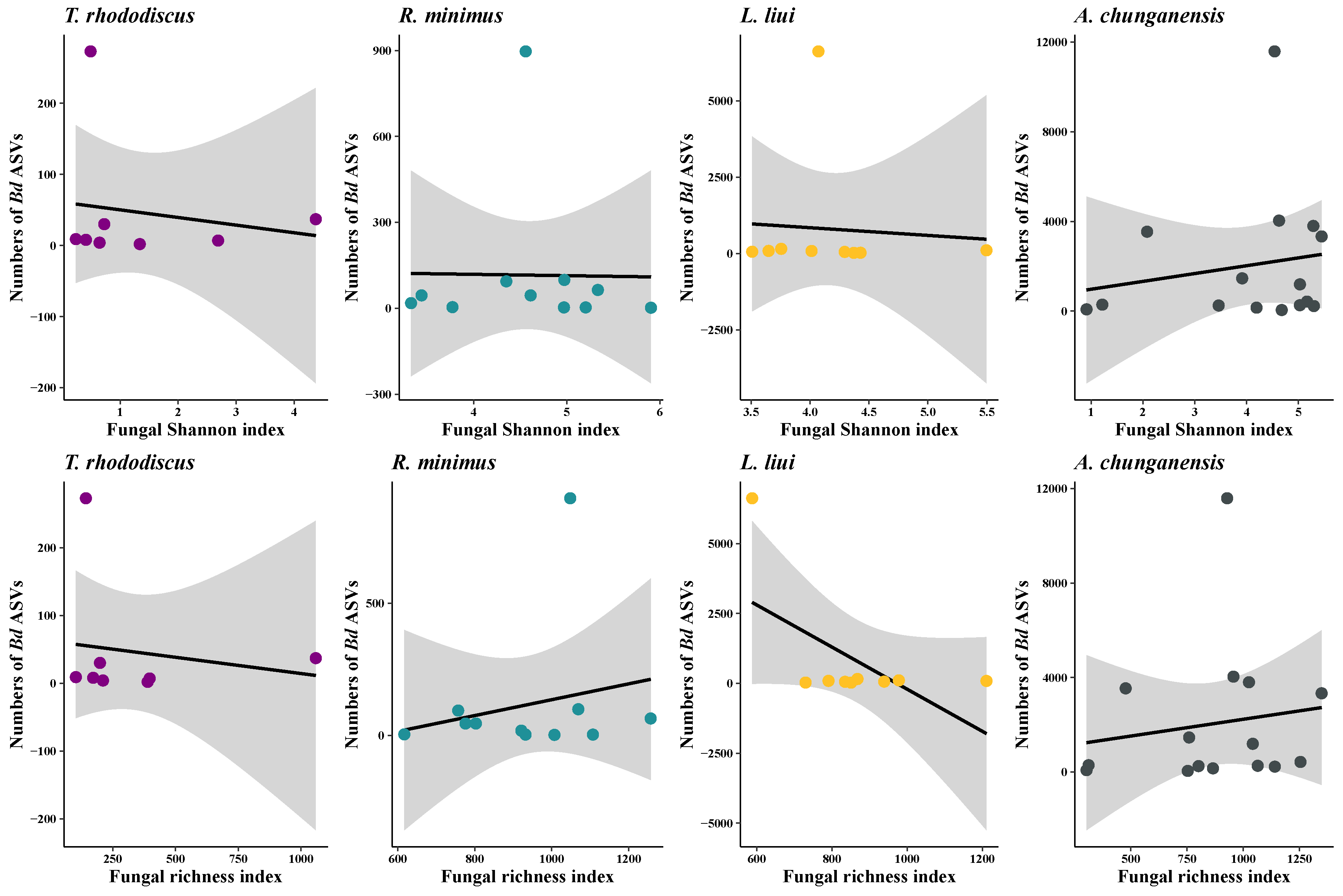


**Supplementary Figure S2**

Linear regressions of fungal alpha diversity values and *Bd* infection intensity (numbers of *Bd* ASVs). Shading represents 95% confidence intervals for each species. Asterisks indicate significant correlations.


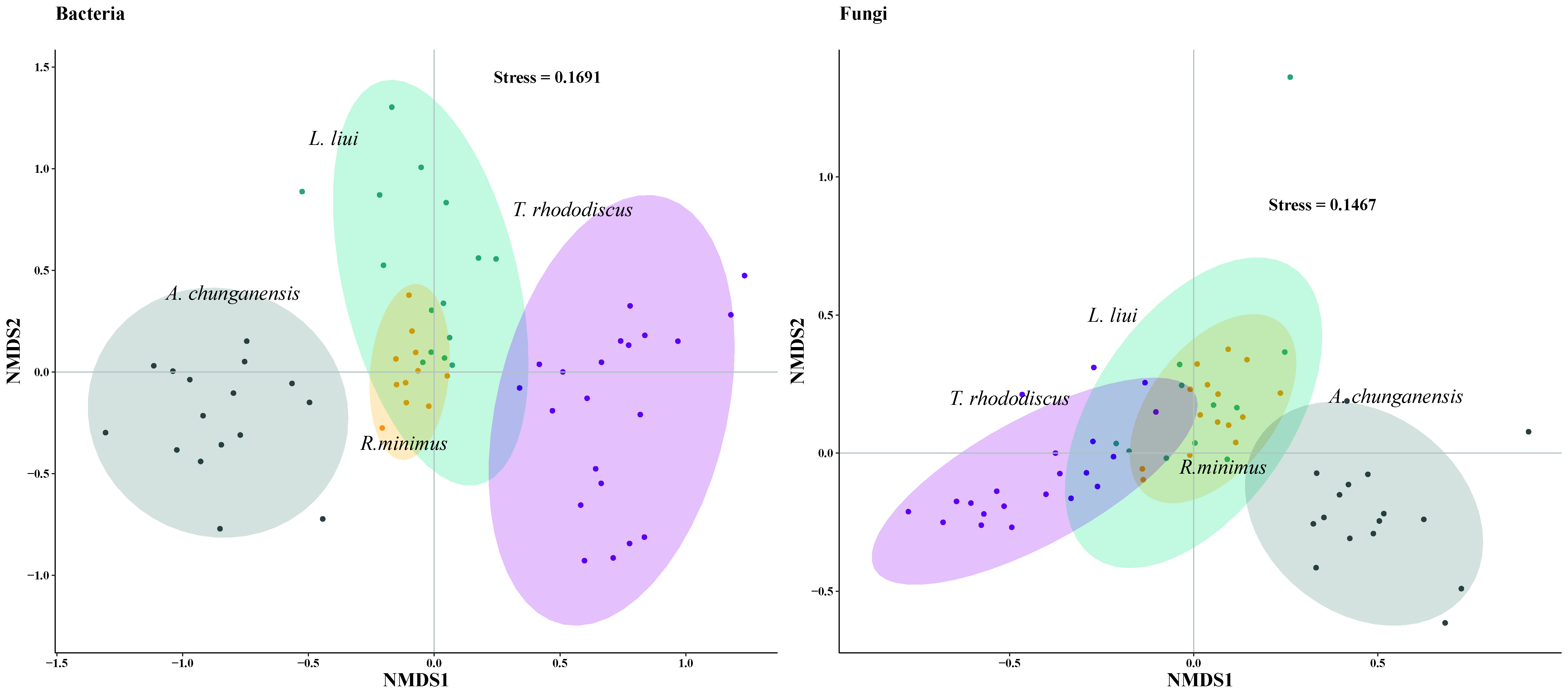


**Supplementary Figure S3**

NMDS of skin bacterial and fungal community compositions based on Bray-Curtis distance across the four host species.


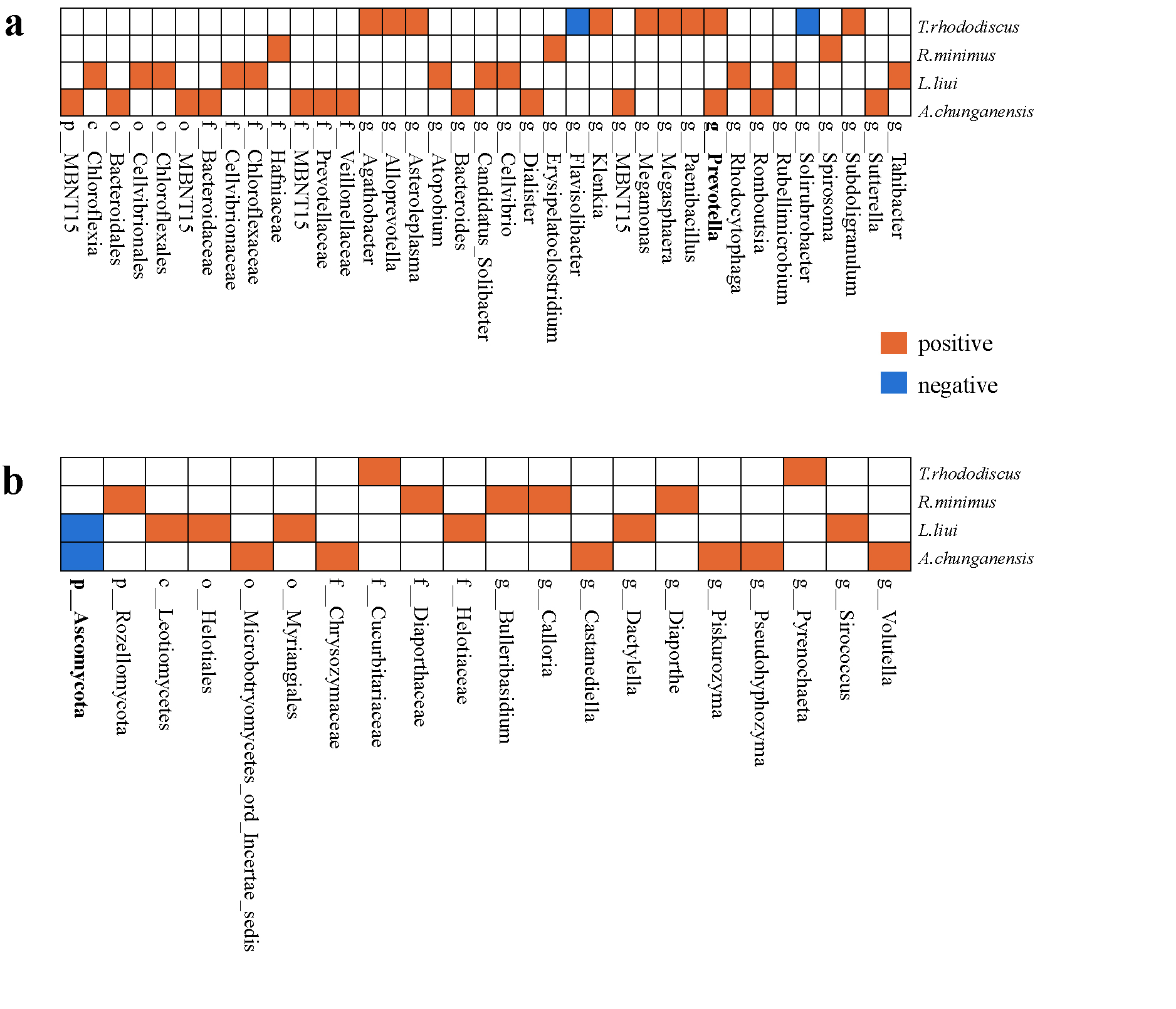


**Supplementary Figure S4**

The significant correlations between bacterial and fungal taxa (phylum, class, order, family, and genus) and *Bd* infection intensity among the four host species.


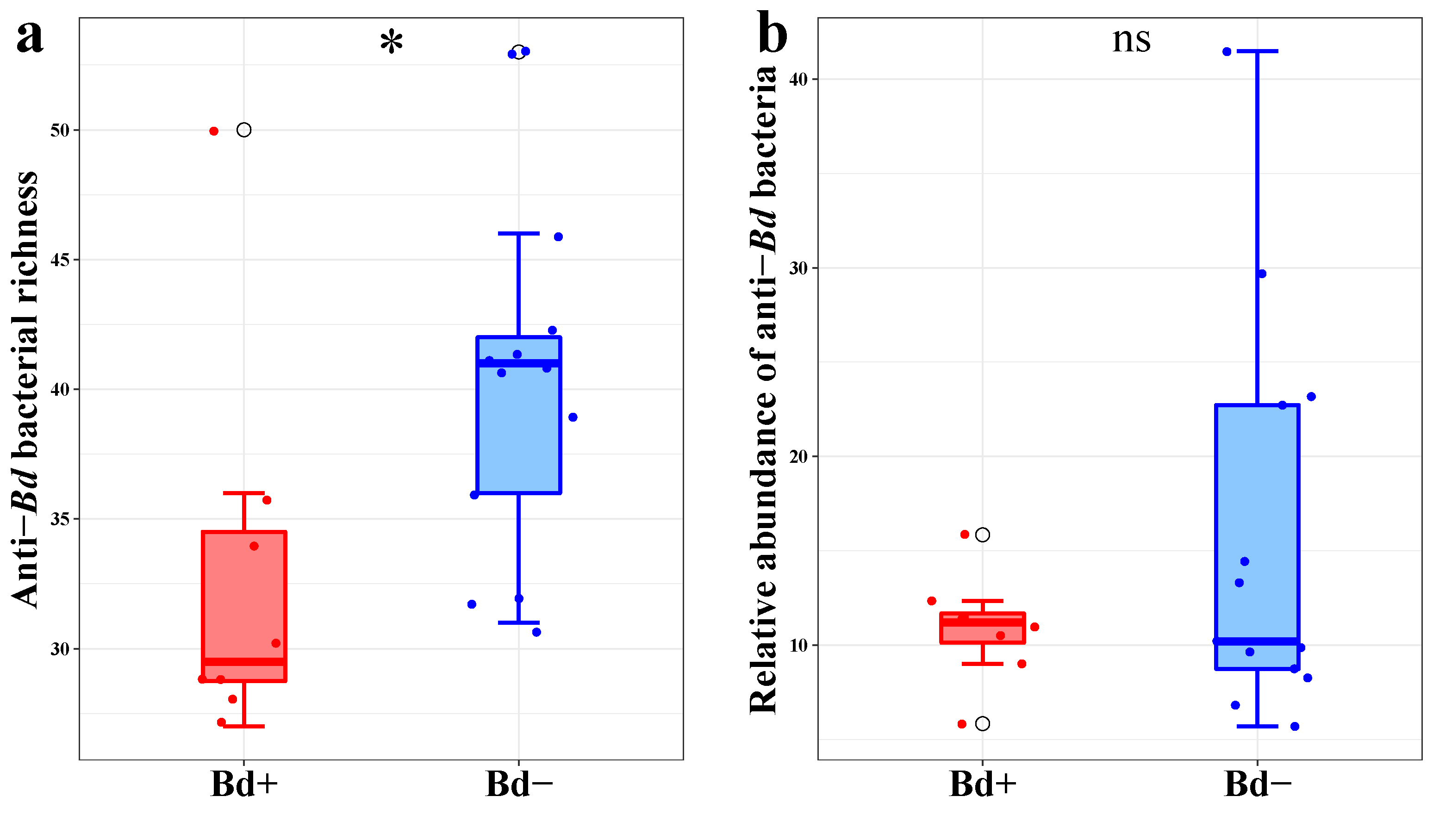


**Supplementary Figure S5**

Putative anti-*Bd* bacterial richness and relative abundance of infected individuals and uninfected individuals of *T. rhododiscus*.
